# The Opponent Channel Population Code of Sound Location Is an Efficient Representation of Natural Binaural Sounds

**DOI:** 10.1371/journal.pcbi.1004294

**Published:** 2015-05-21

**Authors:** Wiktor Młynarski

**Affiliations:** Max-Planck Institute for Mathematics in the Sciences, Leipzig, Germany; University of Tübingen and Max Planck Institute for Biologial Cybernetics, GERMANY

## Abstract

In mammalian auditory cortex, sound source position is represented by a population of broadly tuned neurons whose firing is modulated by sounds located at all positions surrounding the animal. Peaks of their tuning curves are concentrated at lateral position, while their slopes are steepest at the interaural midline, allowing for the maximum localization accuracy in that area. These experimental observations contradict initial assumptions that the auditory space is represented as a topographic cortical map. It has been suggested that a “panoramic” code has evolved to match specific demands of the sound localization task. This work provides evidence suggesting that properties of spatial auditory neurons identified experimentally follow from a general design principle- learning a sparse, efficient representation of natural stimuli. Natural binaural sounds were recorded and served as input to a hierarchical sparse-coding model. In the first layer, left and right ear sounds were separately encoded by a population of complex-valued basis functions which separated phase and amplitude. Both parameters are known to carry information relevant for spatial hearing. Monaural input converged in the second layer, which learned a joint representation of amplitude and interaural phase difference. Spatial selectivity of each second-layer unit was measured by exposing the model to natural sound sources recorded at different positions. Obtained tuning curves match well tuning characteristics of neurons in the mammalian auditory cortex. This study connects neuronal coding of the auditory space with natural stimulus statistics and generates new experimental predictions. Moreover, results presented here suggest that cortical regions with seemingly different functions may implement the same computational strategy-efficient coding.

## Author Summary

Ability to localize the position of a sound source is vital to many organisms, since audition provides information about areas which are not accessible visually. While its importance is undisputed, its neuronal mechanisms are not well understood. It has been observed in experimental studies that despite the crucial role of sound localization, single neurons in the auditory cortex of mammals carry very little information about the sound position. The joint activity of multiple neurons is required to accurately localize sound, and it is an open question how this computation is performed by auditory cortical circuits. In this work I propose a statistical model of natural stereo sounds. The model is based on the theoretical concept of sparse, efficient coding which has provided candidate explanations of how different sensory systems may work. When adapted to binaural sounds recorded in a natural environment, the model reveals properties highly similar to those of neurons in the mammalian auditory cortex, suggesting that mechanisms of neuronal auditory coding can be understood in terms of general, theoretical principles.

## Introduction

The precise role played by the auditory cortex in hearing remains unclear. Before reaching cortical areas, raw sounds undergo numerous transformations in the brainstem and the thalamus. This subcortical processing seems more substantial than in other senses and is a specific property of the auditory system. What computations are performed by the cortex on the output generated by lower auditory regions is a question far from being answered.

One of the issues in functional characterization of the auditory cortex is an apparent lack of specificity. Spiking activity of cortical auditory neurons is modulated by sound features such as pitch, timbre and spatial location [1, 2]. Responses invariant to any of those features seem rare. This interdependence is especially puzzling in the context of extracting spatial information. A number of studies attempted to identify “what” and “where” streams in the auditory system (e.g. [3, 4]). Despite those efforts the existence of a sharp separation of spatial and identity information in the auditory cortex is still not definitely confirmed [5, 6].

Neurons reveal sensitivity to sound position in most parts of the mammalian auditory cortex [7]. Their spatial tuning is quite broad — neural firing can be modulated by sounds located anywhere on the azimuthal plane. While activity of single units does not carry information sufficient to accurately localize sounds, larger numbers of neurons seem to form a population code for sound location [8–11]. These observations strongly differ from assumptions made early in the field that the auditory space is represented by a topographic cortical map, where neighboring units would encode the presence of a sound source at proximal positions [12].

Results described above constitute a conceptual challenge for theoretical models of the auditory cortex and understanding its role in spatial hearing in particular. Nevertheless, a number of candidate roles for this region have been proposed. It has been suggested, for instance, that the main function of the primary auditory cortex (A1) is to process sound features extracted by subcortical structures [13] on multiple time scales. Another theory proposes that the auditory cortex in the cat represents abstract entities (such as a bird song or wind) rather than low-level spectrotemporal features of the stimulus [14]. It is also debated whether auditory cortical areas bear similarities to visual areas, and if yes, what sort of understanding can be gained by combining knowledge about those brain regions [15]. From a theoretical perspective one question seems to be particularly important — is there any general principle behind the functioning of auditory cortex, or does it carry out computations which are task- or modality-specific and therefore not performed in other parts of the brain?

A particularly succesful theoretical framework attempting to explain general mechanisms behind the functioning of the nervous system is provided by the Efficient Coding Hypothesis [16, 17]. It proposes that sensory systems maximize the amount of transmitted stimulus information. Under the additional assumption that a typical stimulus activates only a small fraction of a neuronal population, the hypothesis is known as *sparse coding*[18, 19]. Perhaps the strongest prediction of the efficient coding hypothesis is that the neuronal activity at consecutive stages of sensory processing should become less and less redundant, hence more independent. This prediction has been experimentally tested in the auditory system of the cat. Chechik and colleagues [20] recorded neuronal responses to natural sounds at three levels of the auditory hierarchy — Inferior Colliculus (IC), Medial Genniculate Body (MGB) and A1. They observed that spiking activity was progressively less redundant between single neurons, as quantified using information theoretic measures. This result suggests that audition can be understood using concepts provided by the efficient coding hypothesis.

In order to form an efficient stimulus representation, neuronal codes should reflect regularities present in the sensory environment [21]. This implies that by studying statistics of natural input, one should be able to predict neuronal tuning properties. In audition, this idea has been followed by a number of researchers. Starting at the lowest level of the auditory system, Lewicki and Smith [22, 23] demonstrated that learning a sparse representation of natural sound chunks reproduces shapes of cochlear filters of the cat. A recent extension of this work has suggested that while the auditory nerve may be optimally encoding all sounds it encounters, neurons in the cochlear nucleus may be tuned to efficiently represent particular sound classes [24]. Climbing higher in the auditory hierarchy — Carlson et al [25] have reproduced shapes of spectrotemporal receptive fields (STRFs) in the inferior colliculus by learning sparse codes of speech sounds. The relationship between spectrotemporal tuning of cortical neurons and sparse representation of speech spectrograms were explored by Klein, Koerding and Koenig [26, 27]. More recently, some aspects of the topographic structure of the auditory cortex were shown to emerge from statistics of speech sounds by Terashima and Okada [28]. Terashima and colleagues have also studied the connection between sparse codes of natural sound spectra and harmonic relationships revealed by receptive fields of macaque A1 neurons [29]. Maximizing coding efficiency by learning sparse codes has also lead to emergence of signal representations useful in spatial hearing tasks. Asari et al [30] learned overcomplete dictionaries of monaural spectrograms and demonstrated that this representation allows for the segregation of acoustically overlapping and yet spatially separate sound sources (the “cocktail party problem”). A recent study found that sparse coding of a spectrotemporal representation of binaural sound extracts spatial features invariant to sound identity [31].

As discussed above, a growing body of evidence seems to point to efficient coding as a computational process implemented by the auditory system. To date however, the connection between natural stimulus statistics and auditory spatial receptive fields remains unexplained. It is therefore unclear if spatial computations performed by the auditory cortex are unique to this brain area or whether they can be also predicted in a principled way from a broader theoretical perspective.

The present work attempts to connect spatial computations carried out by the auditory cortex with statistics of the natural stimulus. Here, a hierarchical statistical model of stereo sounds recorded in a real auditory environment is proposed. Based on principles of sparse coding the model learns the spectrotemporal and interaural structure of the stimulus. In the next step, it is demonstrated that when probed with spatially localized sounds, higher level units reveal spatial tuning which strongly resembles spatial tuning of neurons in the mammalian auditory cortex. Additionally, the learned code forms an interdependent representation of spatial information and spectrotemporal quality of a sound. Activity of higher units is therefore modulated by sound’s position and identity, as observed in the auditory system.

This study provides computational evidence that spatial tuning of auditory cortical neurons may be a manifestation of an underlying general design principle — efficient coding. Present results suggest that the role of the auditory cortex is to reduce redundancy of the stimulus representation preprocessed by the brainstem. Representation obtained in this way may facilitate tasks performed by higher brain areas, such as sound localization.

## Results

### Recorded sounds

Binaural sound used to train the model was recorded by a human subject walking freely in a wooded area, in the presence of another speaker. The obtained recording included ambient (wind, flowing stream) and transient environmental sounds (wood cracking, steps) as well as human speech. The auditory scene changed over time due to the motion of the the recorder, the speaker, and changes in the environment. It consisted of multiple sound sources emanating from a diverse set of locations, creating together a complex, natural auditory environment. Please refer to the Methods section for details of the recording.

### Overview of the hierarchical model

**Fig 1:**
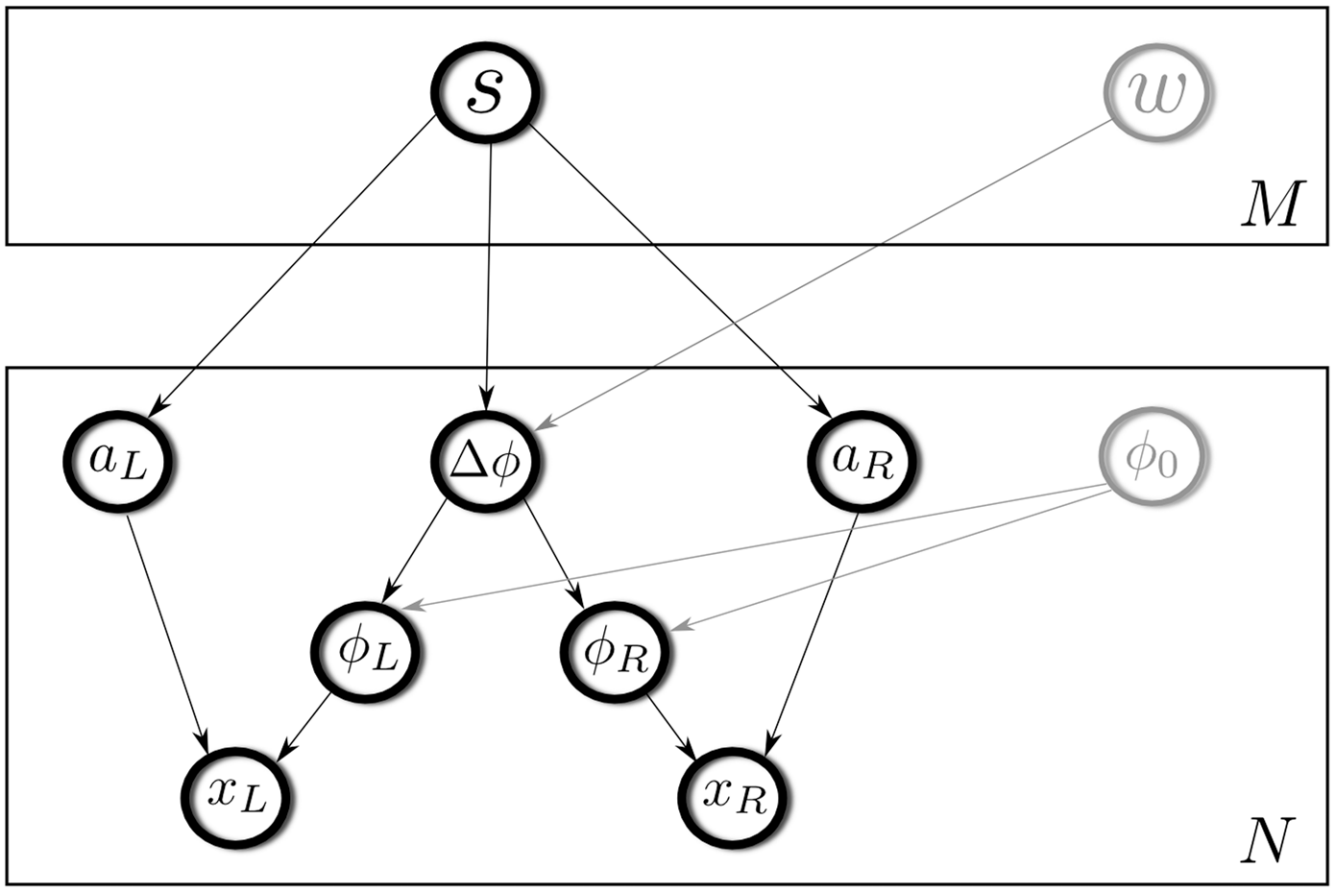
The graphical model representing variable dependencies. The lowest layer represents sound epochs perceived by the left and the right ear *x*_*L*_ and *x*_*R*_. They are decomposed by a sparse coding algorithm into phase and amplitude vectors *ϕ*_*L*_, *ϕ*_*R*_ and *a*_*L*_, *a*_*R*_. Phases are further substracted from each other in order to obtain an IPD vector Δ*ϕ*. The second layer encodes jointly monaural amplitudes and IPDs. Auxiliary variables (phase offset and the scaling factor *w*) are depicted in gray.

The present study proposes a hierarchical statistical model of binaural sounds, which captures binaural and spectrotemporal structure present in natural stimuli. The architecture of the model is shown in Fig 1. It consists of the input layer and two hidden layers. The input to the model was *N* sample-long epochs of binaural sound: from the left ear—*x*_*L*_ and from the right ear—*x*_*R*_. The role of the first layer was to extract and separate phase and amplitude information from each ear by encoding them in an efficient manner. Monaural sounds were transformed into phase (*ϕ*_*L*_, *ϕ*_*R*_) and amplitude (*a*_*L*_, *a*_*R*_) vectors. This layer can be thought of as a statistical analogy to cochlear filtering. Phase vectors were further modified by computing interaural phase differences (IPDs) — a major sound localization cue [32]. This tranformation may be considered an attempt to mimic functioning of the medial superior olive (MSO) — the brainstem nucleus where phase differences are extracted [32].

The second layer of the model learned a joint sparse representation of monaural amplitudes (*a*_*L*_, *a*_*R*_) and phase differences (Δ*ϕ*). Level (amplitude) and temporal (phase) information from each ear was jointly encoded by a population of *M* units. Each of the units was therefore capturing higher-order spectrotemporal patterns of sound in each ear. Additionally, by combining monaural information into single units higher level representation achieved spatial tuning not present in the first layer. The second hidden layer can be interpreted as a model of auditory neurons which receive converging monaural input and jointly operate on phase and amplitude — two kinds of information known to be important for spatial hearing.

### First layer: sparse, complex-valued sound representation

As demonstrated in previous work, filtering properties of the auditory nerve can be explained by sparse coding models of natural sounds [22]. There, short epochs of natural sounds are modelled as a linear combination of real-valued basis functions multiplied by sparse, independent coefficients (i.e. having highly curtotic marginal distributions). Adapted to sets of natural sound chunks, basis functions become localized in time and/or frequency matching properties of cochlear filters.

While being capable of capturing interesting properties of the data, real valued representations are not well suited for modeling binaural sounds. This is because binaural hearing mechanisms utilize interaural level and time differences (ILDs and ITDs respectively). In narrowband channels, differences in time correspond to phase displacements known as interaural phase differences (IPDs). Therefore a desired representation should both be adapted to the data (i.e. non-redundant) and separate amplitude from phase (where phase is understood as a temporal shift smaller than the oscillatory cycle of a particular frequency).

The present work addresses this twofold constraint with complex-valued sparse coding. Each data vector *x* ∈ ℝ^*N*^ is represented in the following way:

$$\begin{array}{c} x_{t}=\sum_{i=1}^{N}R\left\{z_{i}^{*}A_{i,t}\right\}+\eta \end{array}$$ (1)

where *z*_*i*_ ∈ ℂ are complex coefficients, * denotes a complex conjugation, *A*_*i*_ ∈ ℂ^*T*^ are complex basis functions and *η* ∼ 𝓝(0, *σ*) is additive gaussian noise. Complex coefficients in Euler’s form become $z_{i}=a_{i}e^{j\phi_{i}}$ (where $j=\sqrt{-1}$) therefore Eq (1) can be rewritten to explicitely represent phase *ϕ* and amplitude *a* as separate variables:

$$\begin{array}{c} x_{t}=\sum_{i=1}^{N}a_{i}\left(\cos\phi_{i}A_{i,t}^{R}+\sin\phi_{i}A_{i,t}^{I}\right)+\eta \end{array}$$ (2)

Real and imaginary parts $A_{i}^{\text{R}}$ and $A_{i}^{\text{I}}$ of basis functions ${\left\{A_{i}\right\}}_{i=1}^{N}$ span a subspace within which the position of a data sample is determined by amplitude *a*_*i*_ and phase *ϕ*_*i*_. Depending on number of basis functions *N* (each of them is formed by a pair of vectors), the representation can be complete (*N*/2 = *T*) or overcomplete (*N*/2 > *T*).

In a probabilistic formulation, Eqs (1) and (2) can be understood as a likelihood model of the data, given coefficients *z* and basis functions *A*:

$$\begin{array}{c} p\left(x|z,A\right)={\left[\frac{1}{\sigma \sqrt{2\pi }}\right]}^{T}\prod_{t=1}^{T}e^{-\frac{{\left(x_{t}-{\hat{x}}_{t}\right)}^{2}}{2\sigma^{2}}} \end{array}$$ (3)

where ${\hat{x}}_{t}=\sum_{i=1}^{N}\text{R}\{z_{i}^{*}A_{i,t}\}$ is the reconstruction of the *t*−*th* dimension of the data vector *x*. A prior over complex coefficients applied here assumes independence between subspaces and promotes sparse solutions i.e. solutions with most amplitudes close to 0:

$$\begin{array}{c} p\left(z\right)=\frac{1}{Z}\prod_{i=1}^{N}e^{-\lambda S(a_{i})} \end{array}$$ (4)

where *Z* is a normalizing constant. Function *S*(*a*_*i*_) promotes sparsity by penalizing large amplitude values. Here, a Cauchy prior on amplitudes is assumed i.e. $S(a_{i})=log(1+a_{i}^{2})$. One should note however that amplitudes are always non-negative and that in general the Cauchy distribution is defined over the entire real domain. The model attempts to form a data representation keeping complex amplitudes maximally independent across subspaces, while still allowing dependence between coordinates *z* ^𝕽^, *z* ^𝕴^ which determine position within each subspace. Inference of coefficients *z* which represent data vector *x* in the basis *A* is performed by minimizing the following energy function

$$\begin{array}{c} E_{1}\left(z,x,A\right)\propto \frac{1}{2\sigma^{2}}\sum_{t=1}^{T}{\left(\hat{x_{t}}-x_{t}\right)}^{2}+\lambda \sum_{i=1}^{N}S\left(a_{i}\right) \end{array}$$ (5)

which corresponds to the negative log-posterior *p*(*z*∣*x*, *A*). This model was introduced in [33] and used to learn motion and form invariances from short chunks of natural movies. Assuming *N* = *T*/2 and *σ* = 0, it is equivalent to 2-dimensional Independent Subspace Analysis(ISA) [34].

When trained on natural image patches, real and imaginary parts of basis functions *A* form pairs of Gabor-like filters, which have the same frequency, position, scale and orientation. The only differing factor is phase—real and imaginary vectors are typically in a quadrature-phase relationship (shifted by $\frac{\pi }{2}$). By extension, one might expect that the same model trained on natural sounds should form a set of frequency localized phase-invariant subspaces, where imaginary vector is equal to the real one shifted a quarter of a cycle in time. Somewhat surprisingly, such representation does not emerge, and learned subspaces capture different aspects of the data — bandwidth, frequency or time invariance [35, 36].

In order to learn a representation from the statistics of the data that preserves a desired property such as phase invariance, one could select a parametric form of basis functions and adapt the parameter set [37]. Such a parametric approach has the disadvantage that the assumed family of solutions might not be flexible enough to efficiently span the data space. Another, more flexible alternative to learn a structured representation is to regularize basis functions by imposing temporal-coherence promoting priors [36]. This, however, requires determining the strength of regularizing priors.

**Fig 2:**
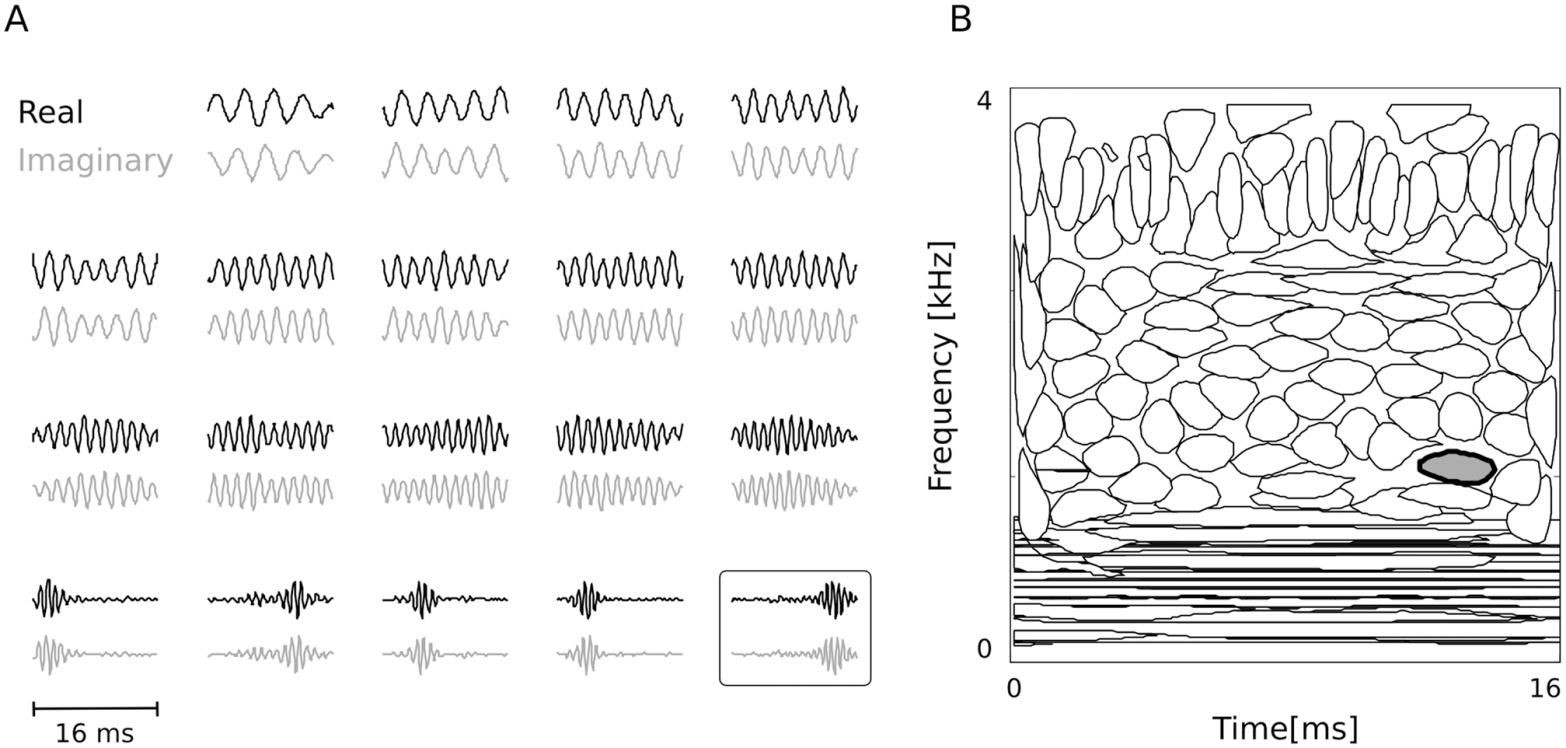
First layer basis. A) Representative real (black) and imaginary (gray) vectors. B) Isoprobability contours of Wigner-Ville distributions associated with each real vector. Time—frequency plane is tiled uniformly with a weak overlap. Gray-filled oval corresponds to the framed basis function on panel A.

To overcome these problems, a different approach was taken here. The first-layer representation was created in two steps. Firstly a real-valued sparse code was trained (see Methods). Learned basis functions were well localized in time or frequency and tiled the time-frequency plane in a uniform and non-overlapping manner (Fig 2B). They were taken as real vectors *A* ^ℜ^ of complex basis functions *A*. In the second step, imaginary parts were created by performing the Hilbert transform of real vectors. The Hilbert transform of a time varying signal *y*(*t*) is defined as follows:

$$\begin{array}{c} H\left(y\left(t\right)\right)=\frac{1}{\pi }p.v.\int_{-\infty }^{\infty }\frac{y(\tau )}{t-\tau }d\tau \end{array}$$ (6)

Where *p*.*v*. stands for Cauchy principal value. In such a way every real vector $A_{i}^{ℜ}$ was paired with its Hilbert transform $A_{i}^{ℑ}=H(A_{i}^{ℜ})$ i.e. a vector which complex Fourier’s coefficients are all shifted by $\frac{\pi }{4}$ in phase. The obtained dictionary is adapted to the stimulus ensemble, hence providing a non-redundant data representation, yet makes phase clearly interpretable as a temporal displacement.

The model was trained using *T* = 128 sample-long chunks of sound sampled at 8 kHz, which corresponds to 16 ms duration. The complete representation of 128 real basis functions was trained, and each of them was paired with its Hilbert transform, resulting in the total number of 256 basis vectors. Selected basis functions are displayed in Fig 2A. Real vectors are plotted in black together with associated imaginary ones plotted in gray. Panel B of the same figure displays isoprobability contours of Wigner-Ville distributions associated with the 256 basis functions. This form of representation localizes each temporal feature on a time-frequency plane [38] (one should note that real and imaginary vectors within each pair are represented by the same contour on that plot). A clear separation into two classes is visible. Low frequency basis functions (below 1 kHz) are non-localized in time (spanning the entire 16 ms interval), while in higher frequency regions their temporal precision increases. An interesting bandwidth reversal is visible around 3 kHz, where temporal accuracy is traded for frequency precision. Interestingly, the sharp separation into frequency and time localized basis functions, which emerged in this study was not clearly visible in other studies which performed sparse coding of sound [22, 38]. Time-frequency properties observed here reflect the statistical structure of the recorded auditory scene, which mostly consisted of non-harmonic environmental sounds sparsely interspersed with human speech.

**Fig 3:**
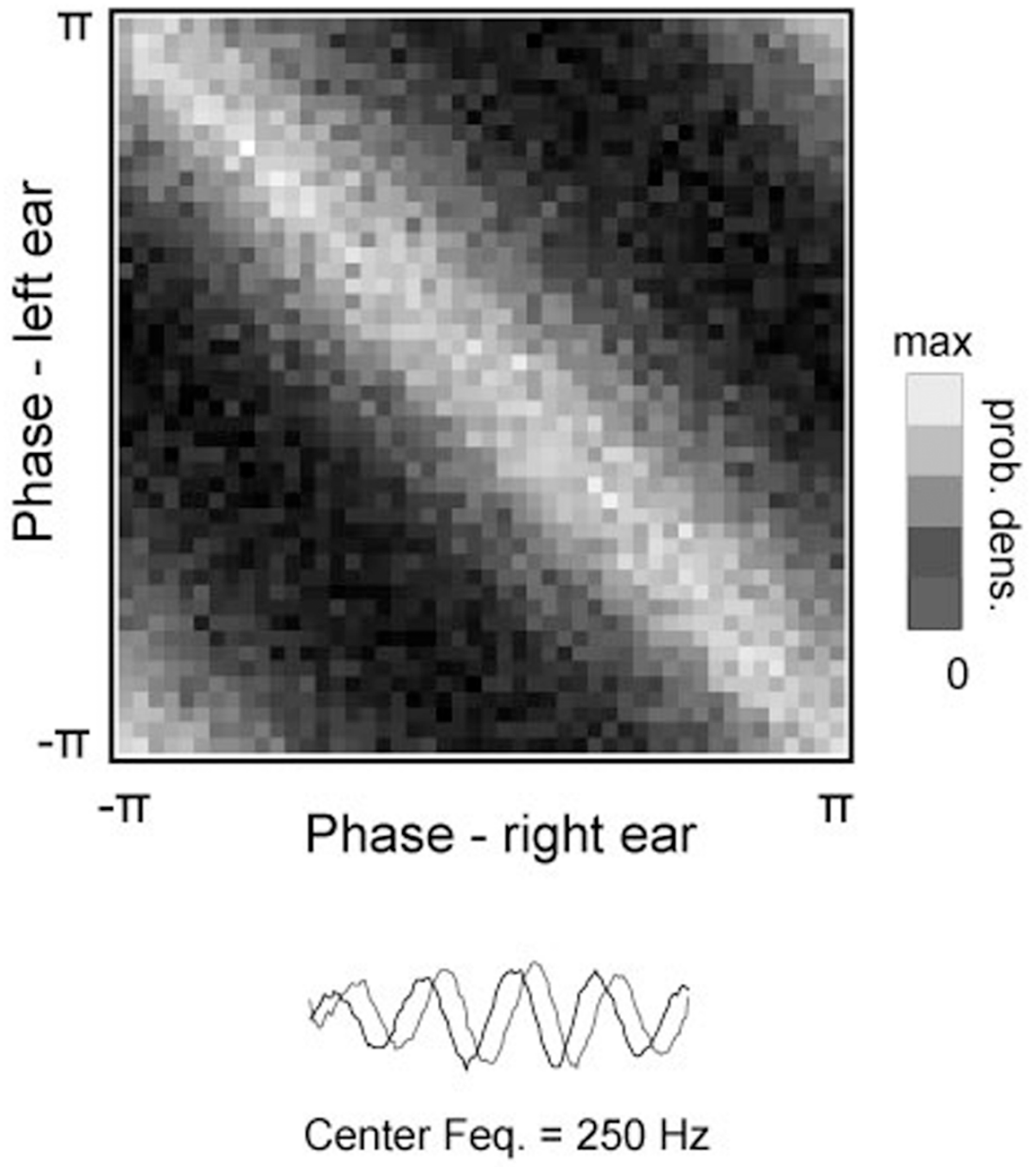
Joint distribution of monaural phases. The distribution was estimated by independently encoding left and right ear sounds from an ensemble of binaural sound epochs and creating a histogram of phase values associated with the basis function depicted at the bottom of the figure. Visible ridge-like pattern implies that monaural phases reveal a dependence in their difference.

Fig 3 depicts a typical distribution of binaural phase. Phases of the same basis function in each ear reveal dependence in their difference. This means that joint probability of monaural phases depends solely on the IPD:

$$\begin{array}{c} p\left(\phi_{i,L},\phi_{i,R}\right)\propto p\left(\Delta \phi_{i}\right) \end{array}$$ (7)

where Δ*ϕ*_*i*_ = *ϕ*_*i*, *L*_−*ϕ*_*i*, *R*_ is the IPD. This property is a straightforward consequence of physics of sound — sounds arrive to each ear with a varying delay giving rise to positive and negative phase shifts. From a statistical point of view this means that monaural phases become conditionally independent given their difference and a phase offset *ϕ*_*i*, *O*_:

$$\begin{array}{c} \phi_{i,L}⊥\phi_{i,R}|\Delta \phi_{i},\phi_{i,O} \end{array}$$ (8)

The phase offset *ϕ*_*i*, *O*_ is the absolute phase value — indicating the time from the beginning of the oscillatory cycle. It can be therefore said that:

$$\begin{array}{c} \phi_{i,L}=\phi_{i,O}+\frac{\Delta \phi_{i}}{2} \end{array}$$ (9)

$$\begin{array}{c} \phi_{i,R}=\phi_{i,O}-\frac{\Delta \phi_{i}}{2} \end{array}$$ (10)

This particular statistical property allows us to understand IPDs not as an ad-hoc computed feature but as an inherent property of the probability distribution underlying the data. It is reflected in the structure of the graphical model (see Fig 1). Since the phase offset *ϕ*_*i*, *O*_ does not carry spatial information for the purposes of current study it is treated as an auxiliary variable and therefore marked in gray.

### Second layer: joint representation of monaural amplitudes and interaural phase differences

In an approach to model the cochlear coding of sound, monaural sound epochs *x*_*L*_ and *x*_*R*_ were encoded independently using the same dictionary of complex basis functions *A* described in the previous section. Signal from both ears converged in the second hidden layer, which role was to form a joint, higher-order representation of the entire stimulus processed by the auditory system.

The celebrated Duplex Theory of spatial hearing specifies two kinds of cues used to solve the sound-localization task: interaural level and time (or phase) differences [39]. While IPDs are supposed to be mostly used in localizing low-frequency sounds, ILDs are a cue, which (at least in the laboratory conditions) can be used to identify the position of high frequency sources. Phase and level cues are known to be computed in lateral and medial superior olive (LSO and MSO respectively) — separated anatomical regions in the brainstem [32]. However, an assumption made here was that neurons in the auditory cortex receive converging input from subcortical structures. This would enable them to form their spatial sensitivity using both fine structure phase and amplitude information. One can take also the inverse perspective: a single object (a “cause”) in the environment generates level and phase cues at the same time. Its identification therefore has to rely on observing dependencies between those features of the stimulus.

The second layer formed a joint representation of monaural amplitudes and interaural phase differences. However, not all IPDs were modelled in that stage. Humans stop utilizing fine structure IPDs in higher frequency regimes (roughly above 1.3 kHz), since this cue becomes ambiguous [32]. Aditionally, cues above around 700 Hz become ambiguous (a single cue value does not correspond to a unique source position). For those reasons, and in order to reduce the number of data dimensions, 20 out of 128 IPD values were selected. The selection criteria were the following: (i) an associated basis function should have the peak of the Fourier spectrum below 0.75 kHz (which provided the upper frequency bound), and (ii) it should have at least one full cycle (which provided the lower bound). All basis functions fulfilling these criteria were non-localized in time (they spanned entire 16 ms interval). As a result, the second layer of the model was jointly encoding *T* = 128 log-amplitude values from each ear and *P* = 20 phase differences.

Monaural log-amplitude vectors *a*_*L*_, *a*_*R*_ ∈ ℝ^*T*^ were concatenated into a single vector *a* ∈ ℝ^2×*T*^, and encoded using a dictionary of amplitude basis functions *B*. Representation of IPDs (Δ*ϕ*) was formed using a separate feature dictionary *ξ*. Both — phase and amplitude basis functions (*B* and *ξ*), were coupled by associated sparse coefficients *s*. The overall generative model of phases and amplitudes was defined in the following way:

$$\begin{array}{c} a_{n}=\sum_{i=1}^{M}s_{i}B_{i,n}+\eta \end{array}$$ (11)

$$\begin{array}{c} \Delta \phi_{n}=\left|w\right|\sum_{i=1}^{M}s_{i}\xi_{i,n}+\epsilon \end{array}$$ (12)

The amplitude noise was assumed to be gaussian (*η* ∼ 𝓝(0, *σ*_2_)) with *σ*_2_ variance. Since phase is a circular variable its noise *ε* was modelled by the von Mises distribution with concentration parameter *κ*.

The second layer was encoding two different physical quantities — phases, which are circular values, and log-amplitudes, which are real numbers. The goal was to form a joint representation of both parameters and learn their dependencies from the data. A simple, linear sparse coding model could be in principle used to achieve this task. However, if a single set of sparse coefficients *s*_*i*_ was used to model both quantities, scaling problems could arise, namely a coefficient value which explains well the amplitude vector may be too large or too small to explain the concomittant IPD vector. For this reason an additional phase multiplier *w* was introduced. It enters Eq 11 as a scaling factor, which gives the model additional flexibility required to learn joint probability distribution of amplitudes and IPDs. Fig 1 depicts it in gray as an auxiliary variable. In this way, amplitude values and phase differences were modelled by variables sharing a common, sparse support (coefficients *s*), with a sufficient flexibility.

Pairs of basis functions *B*_*i*_, *ξ*_*i*_ represent binaural spectrotemporal stimulus and IPD patterns respectively, while sparse coefficients *s* signal their joint presence in the encoded sound epoch. An *i*−*th* second-layer unit was activated (*s*_*i*_ ≠ 0) whenever a pattern of IPDs represented by the basis function *ξ*_*i*_ or a pattern of amplitudes represented by *B*_*i*_ was present in its receptive field. The activity was maximized, when both features were present at the same time. For this reason, when seeking analogies between the higher-level representation and auditory neurons, coefficients *s* can be interpreted as neuronal activity (e.g. firing rate) and basis function pairs *B*_*i*_, *ξ*_*i*_ as receptive fields (i.e. stimulus preferred by a neuron).

The likelihood of amplitudes and phase differences defined by the second layer was given by:

$$\begin{array}{c} p\left(a,\Delta \phi |s,w,B,\xi \right)={\left[\frac{1}{\sigma_{2}\sqrt{2\pi }}\right]}^{2T}\prod_{n=1}^{2T}e^{-\frac{{\left(a_{n}-{\hat{a}}_{n}\right)}^{2}}{2\sigma_{2}^{2}}}\;{\left[\frac{1}{2\pi I_{0}\left(\kappa \right)}\right]}^{P}\prod_{m=1}^{P}e^{\kappa \cos(\Delta \phi_{m}-{\hat{\Delta \phi }}_{m})} \end{array}$$ (13)

where ${\hat{a}}_{n}=\sum_{i=1}^{M}s_{i}B_{i,n}$, ${\hat{\Delta \phi }}_{m}=|w|\sum_{i=1}^{M}s_{i}\xi_{i,m}$ are amplitude and phase reconstructions repsectively and *I*_0_ is the modified Bessel function of order 0. The joint distribution of coefficients *s* was assumed to be equal to the product of marginals:

$$\begin{array}{c} p\left(s\right)=\frac{1}{Z}\prod_{i=1}^{M}e^{-\lambda_{2}S\left(s_{i}\right)} \end{array}$$ (14)

where *λ*_2_ is a sparsity controlling parameter. A Cauchy distribution was assumed as a prior over marginal coefficients (i.e. $S(s_{i})=\log(1+s_{i}^{2})$). To prevent degenerate solutions, where sparse coefficients *s* are very small and the scaling coefficient *w* grows undbounded, a prior *p*(*w*) constraining it from above and from below was placed. A generalized Gaussian distribution of the following form was used:

$$\begin{array}{c} p\left(w\right)=\frac{\beta }{2\alpha \Gamma (\frac{1}{\beta })}e^{-{\left(\frac{\left|w-\mu \right|}{\alpha }\right)}^{\beta }} \end{array}$$ (15)

Γ denotes tha gamma function, *α*, *β* and *μ* denote the scale, shape and location parameters respectively. When the shape parameter *β* is set to a large value (here *β* = 8), the distribution approximates a uniform distribution. Varying the scale parameter *α* changes the upper and the lower limit of the interval.

Taken together the negative log-posterior over the second layer coefficients was defined by the energy function:

$$\begin{array}{c} E_{2}\left(s,w,B,\xi \right)\propto \frac{1}{\sigma_{2}^{2}}\sum_{n=1}^{2\times T}{\left(a_{n}-{\hat{a}}_{n}\right)}^{2}+\kappa \sum_{m=1}^{P}\cos\left(\Delta \phi_{m}-{\hat{\Delta \phi }}_{m}\right)+\lambda_{2}\sum_{i=1}^{M}S\left(s_{i}\right)+\lambda_{w}{\left(\frac{\left|w-\mu \right|}{\alpha }\right)}^{\beta } \end{array}$$ (16)

the *λ*_*w*_ coefficient was introduced to control the strength of the prior on the scaling coefficient w. Similarly as in the first model layer, learning of basis functions and inference of coefficients was performed using gradient descent (see Methods). The total number *M* of basis function pairs was set to 256.

## Properties of the second layer representation

**Fig 4:**
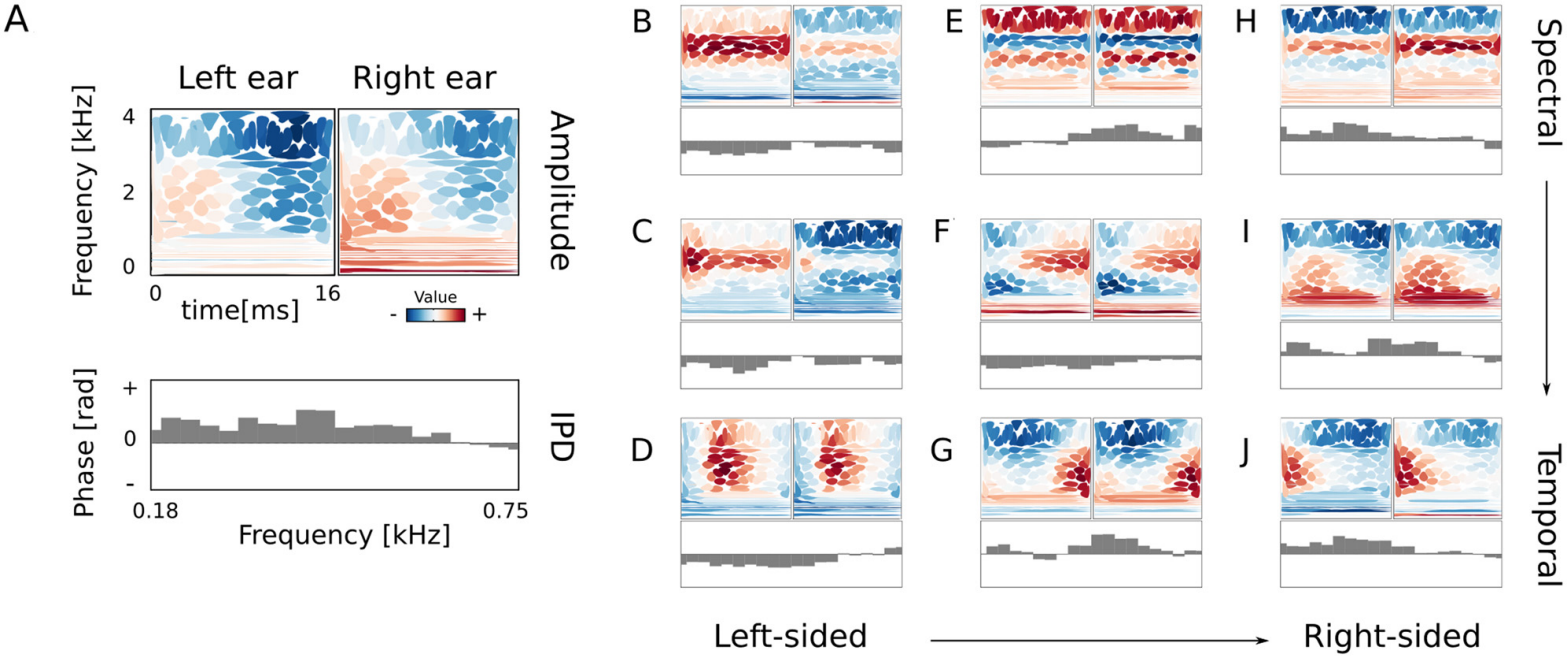
Higher-order basis functions. A) Explanation of the visualization of second layer basis functions. Top two panels depict the binaural amplitude basis function *B*_*i*_. Spectrotemporal information in each ear is represented using isoprobability contours of Wigner-Ville distributions of first-layer basis functions (see Fig 2). Colors correspond to the log-amplitude weight. The bottom panel represents the IPD basis function *ξ*_*i*_. Each gray bar represents one of 20 selected low-layer basis functions. Here almost all values are positive (the bars point upwards), which corresponds to the right-ear precedence. B)-J) Basis functions ordered vertically by spectral modulation and horizontally by the dominating side.

The second layer learned cooccuring phase and amplitude patterns forming a sparse, combinatorial code of the first layer output. Fig 4 displays 10 representative examples of basis function pairs *ξ*_*i*_ and *B*_*i*_, which encoded amplitudes and IPDs respectively.

Each amplitude basis function consisted of two monaural parts corresponding to the left and right ear. First-layer, temporal features were visualized using contours of Wigner-Ville distribution and colored according to the relative weight. Entries of IPD basis functions were values (marked by gray bars) modelling interaural phase disparities in each of selected 20 frequency channels.

The subset of 9 basis functions depicted in subpanels B-J of Fig 4 constitutes a good representation of the entire dictionary. Their vertical ordering corresponds to spectrotemporal properties of *B*_*i*_ basis functions. Amplitude features displayed in the first row (B, E, H) reveal pronounced spectral modulation, while the last row (D, G, J) are features which are strongly temporaly modulated. Columns are ordered according to the ear each basis function pair prefered. Left column (B, C, D) are left-sided basis functions. Higher amplitude values are visible in the left ear parts (although differences are rather subtle), while associated IPD features are all negative. IPDs smaller than 0 imply that the encoded waveform was delayed in the right ear, hence the sound source was closer to the left ear. The last column (H, I, J) depicts more right-sided basis functions. Features displayed in the middle column (E, F, G) weight binaural amplitudes equally, however entries of associated phase vectors are either mostly negative or mostly positive.

As Fig 4 shows, higher level representation learned spectrotemporal properties of the auditory scene, reflected in shapes of amplitude basis functions *B*_*i*_. Binaural relations were captured by relative weighting of amplitudes in both ears and the shape of the IPD basis function.

**Fig 5:**
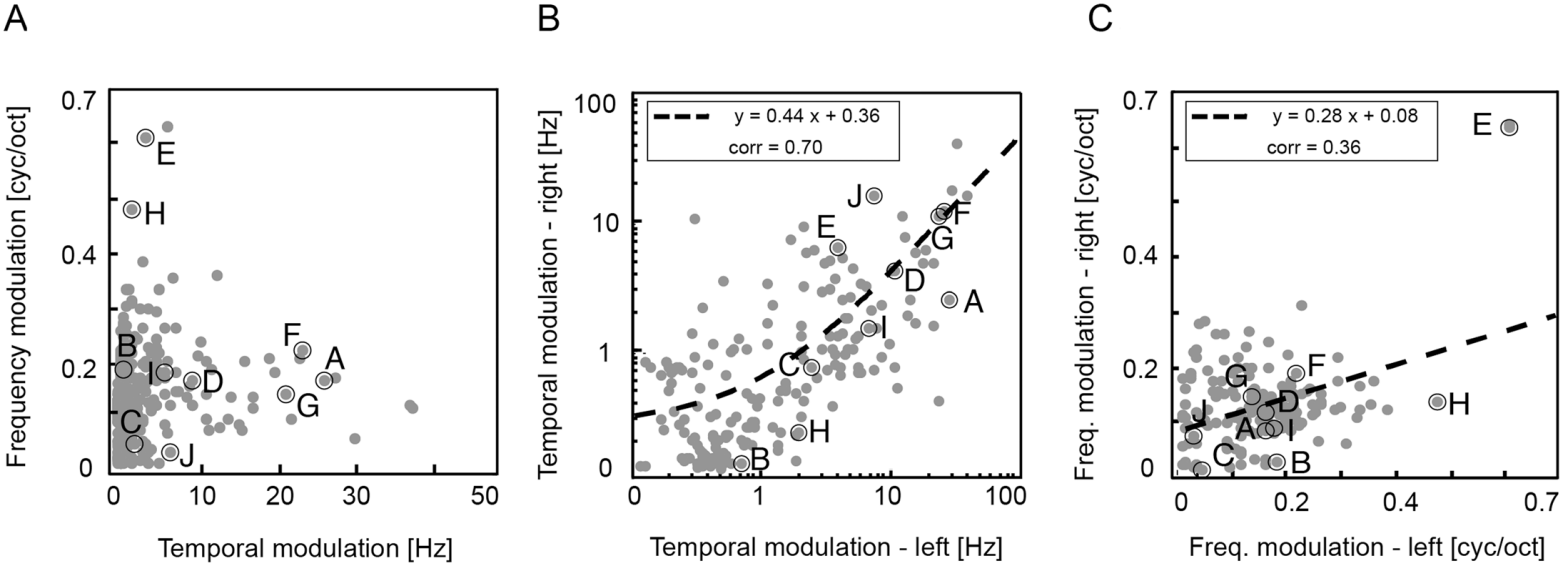
Spectrotemporal properties of the representation. A) Centers of mass of monaural modulation spectra. B) Centers of mass of temporal modulation in monaural parts of *B*_*i*_ basis functions plotted C) Centers of mass of spectral modulation in monaural parts of *B*_*i*_ basis functions plotted. Letters correspond to panels in Fig 4. Black dashed lines depict linear regression fits. Parameters of each fit are written in figure insets.

To get a more detailed understanding of the spectrotemporal features captured by the representation, analysis of modulation spectra was performed. A modulation spectrum is a 2D Fourier transform of the spectrotemporal representation of a signal. It is known that modulation spectra of natural sounds posess specific structure [40]. Here, modulation spectrum was computed separately for monaural parts of amplitude basis functions *B*_*i*_ (see Methods). In the next step a center of mass of each of the modulation spectra was computed. Centers of mass are represented by single points in Fig 5A.

A clear tradeoff between spectral and temporal modulation was visible. Basis functions which were strongly temporally modulated revealed simultaneously weak spectral modulation (and vice versa). It is evident as a “triangular” shape of the point distribution in Fig 5A. This seems to be a robust property of natural sounds [40] and was shown to be captured by sparse coding models [25–27, 41, 42]. Interestingly, spectro-temporal receptive fields of auditory neurons share this property [43, 44]. Auditory neurons which reveal sensitivity to spectrotemporal sound patterns seem to prefer sounds which are either modulated in time or in frequency, but not both. When compared with modulation spectra of natural sound this form of tuning may be understood as an adaptation to the environmental stimulus statistics.

Average temporal modulation in the left ear is plotted against the right ear in panel B. As visible — the amplitude modulation of basis functions B varied between 0 and 40 Hz, and a general linear trend was present. A linear regression model was fitted to these data (the fit is depicted in Fig 5 as a black dashed line). The fit has revealed a relatively strong linear relationship between the temporal variation of monaural parts (with Pearson’s correlation *ρ* = 0.70).

Spectral amplitude modulation revealed a weaker interaural correlation (*ρ* = 0.36). This is visible in Fig 5C—points representing amplitude basis functions are scattered stronger than in panel B of the same figure. This property can be explained by head filtering characteristics. Acting as a low-pass filter, the head attenuates higher frequencies. For this reason, fine spectral information above 1.5 kHz was typically more pronounced in a single ear, affecting the strength of spectral modulation. This may be considered as an example of how stimulus statistics are determined not only by the environmental properties, but also by the anatomy of an organism. The majority of basis functions revealed spectral modulation smaller than 0.4 cycle per octave, and only a single one exceeded this value.

**Fig 6:**
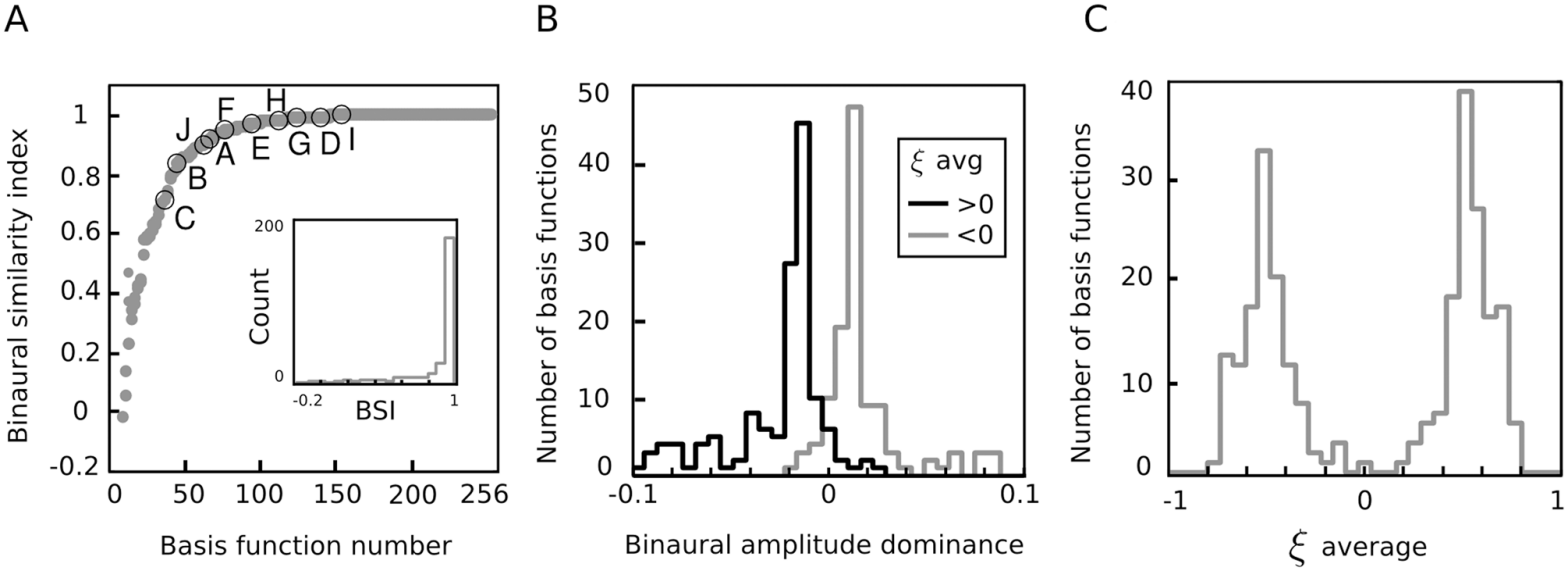
Binaural properties of the representation. A) Binaural Similarity Index of amplitude basis functions *B*_*i*_. The BSI is a correlation coefficient between left and right ear subvectors. The inset depicte the BSI histogram. B) Distribution of binaural amplitude dominance. Values above 0 imply domination of the left, and below 0 of the right ear. Histograms of BAD values of amplitude basis functions associated with negative IPD basis functions are colored gray and those associated with positive *ξ*_*i*_ values are colored black. C) Distribution of averages of normalized *ξ*_*i*_ basis functions.

In the following analysis step, the goal was to analyze similarity in the monaural spectrotemporal patterns encoded by each second-layer unit. To this end binaural similarity index (BSI) of each amplitude basis function [43] was computed. The BSI is a correlation coefficient between the left and the right parts of a binaural, spectrotemporal feature. If the BSI was close to 0, the corresponding unit was representing different spectrotemporal patterns in each ear, while values close to 1 implied high similarity. BSIs are plotted in Fig 6A.

Clearly, an overwhelming majority of basis functions revealed high interaural similarity (*BSI* > 0.8, see the histogram at the inset). BSI of only one basis function was slightly below 0. If information encoded by amplitude basis functions in each ear was independent, the BSI distribution should peak at 0. This observation suggests that most of the second-layer units captured the same “cause” underlying the stimulus i.e. a binaurally redundant spectrotemporal pattern. While the BSI index measures similarity of encoded monaural sound features, it is not informative about the side-preference of each unit. To asess whether amplitude basis functions were biased more towards the left or towards the right ear, another statistic — a binaural amplitude dominance (BAD) was computed. The amplitude dominance was defined in the following way:

$$\begin{array}{c} BAD\left(B_{i}\right)=\log(\frac{∥\exp(B_{i,L})∥}{∥\exp(B_{i,R})∥}) \end{array}$$ (17)

where *B*_*i*, *L*_ = *B*_*i*, (1, …, *T*)_, *B*_*i*, *R*_ = *B*_*i*, (*T*+1, …,2×*T*)_ are left and right ear parts of an amplitude basis function *B*_*i*_. Each of them was pointwise exponentiated to map the entries from real log-amplitude values to the positive amplitude domain. The BAD index value larger than 0 means that the left-ear amplitude vector had a larger norm (i.e., it dominated the input to the particular unit). Balanced units had a BAD value close to 0 while right-ear dominance was indicated by negative values. Two histograms of dominance scores are displayed in panel B of Fig 6. The black one is an empirical distribution of BAD values of amplitude basis functions associated with IPD features of a negative average value (left-side preferring). The gray one corresponds to amplitude features matched with right-side biased phase basis functions. Both distributions are roughly symmetric with their modes located quite close to 0. Such bimodal distribution of the amplitude dominance score implies that amplitude basis functions could be divided into two opposite populations — each preferring input from a different ear. Moreover, amplitude and phase information modelled by basis functions *B*_*i*_ and *ξ*_*i*_ was dependent — amplitude features dominated by information from one ear were associated with IPD features biased towards the same ear.

While amplitude representation encoded the quality of the sound together with binaural differences, the IPD dictionary was representing solely spatial aspects of the stimulus i.e. the temporal difference between the ears. In almost entire feature population, single entries of each of the phase difference basis functions *ξ*_*i*_ all had the same sign. Negative phase differences corresponded to the left-side bias (it meant that the soundwave arrived first to the left-ear generating a smaller phase value) and positive to the right-side one. These two properties allowed us to asess the spatial preference of IPD basis functions simply by computing the average of their entries. The histogram of averages of vectors *ξ*_*i*_ (normalized to have the maximal absolute value of 1) is depicted in Fig 6C. A clear bimodality is visible in the distribution. The positive peak corresponds to right-sided basis functions and the negative one to the left-sided subpopulation. Almost no balanced features (close to 0) were present in the dictionary. This dichotomy is visible also in Fig 4—binaurally balanced amplitude basis functions (middle column) were associated with phase vectors biased towards either side. This result may be related to a previous study, which showed that a representation of natural IPD distribution designed to maximize stimulus discriminability (Fisher information) also has a form of two distinct channels [45], where each of the channels preferred IPDs of an opposite sign.

### Spatial tuning of second layer units

The second layer of the model learned a distributed representation of sound features accesible to neurons in the auditory cortex. Assuming that the cortical auditory code indeed develops driven by principles of efficiency and sparsity, one can interpret second layer basis functions as neuronal receptive fields and sparse coefficients *s* as a measure of neuronal activity (e.g. firing rates). The model can be then probed using spatial auditory stimuli. If it indeed provides an approximation to real neuronal computations, its responses should be comparable with spatial tuning properties of the auditory cortex.

**Fig 7:**
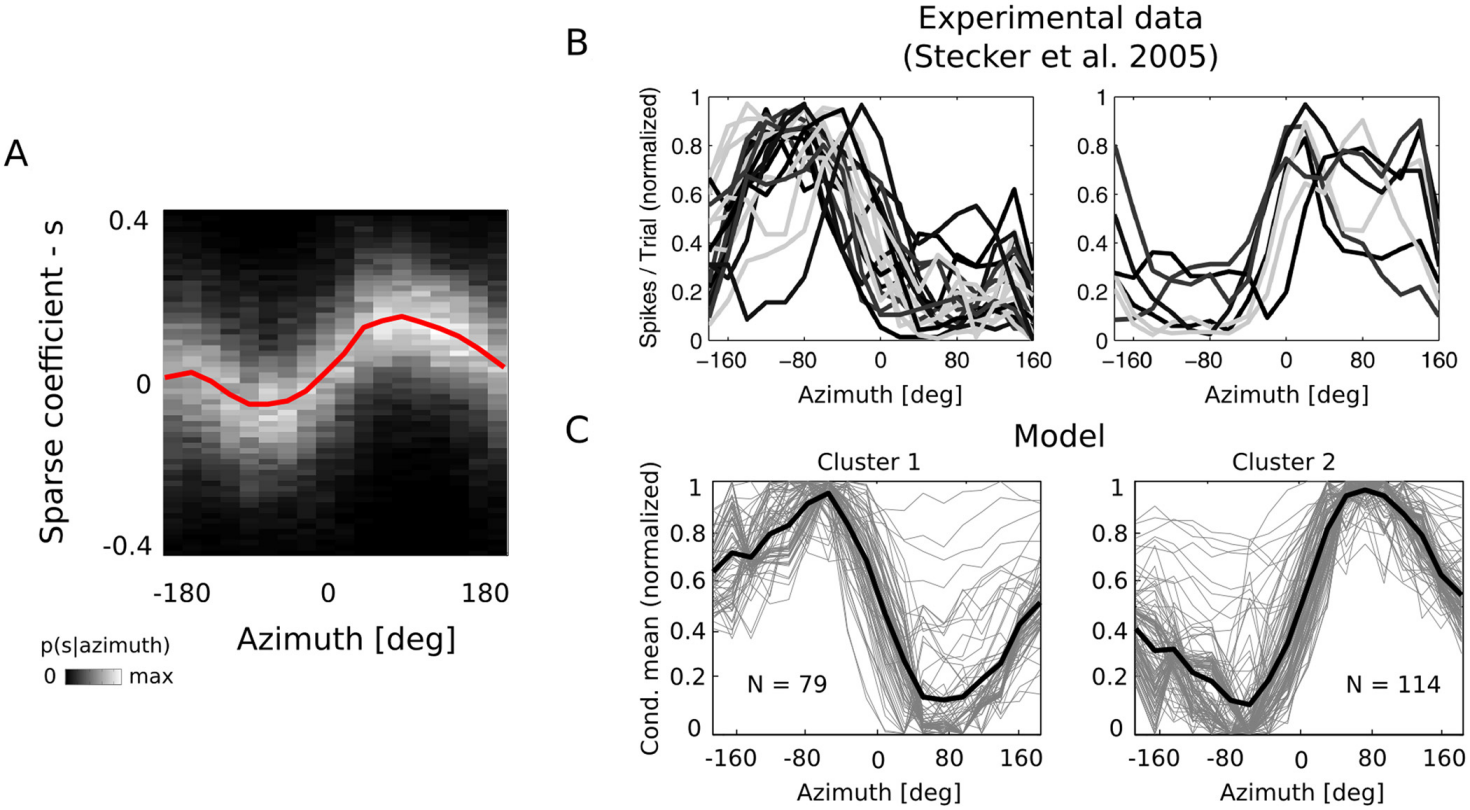
Spatial tuning curves of second-layer units. A) Conditional distribution of the coefficient *s*_*i*_ corresponding to basis functions *B*_*i*_, *ξ*_*i*_ depicted in Fig 4A. The red line depicts the average value conditioned in sound position. B) Experimentally measured spatial tuning curves measured in the A1 area of the cat. The left panel depicts contra- and the right panel ipsi- laterally tuned units. Figure modified from [8] C) All position-modulated tuning curves belonging to each of the two clusters. Thin gray lines are single tuning curves, while thick black lines depict cluster averages.

In order to verify whether this was true, a test recording was performed. As a test sound the hiss of two pieces of paper rubbed against each other was used. It was a broadband signal, reminiscent of white noise used in physiological experiments, yet posessing natural structure. Recording was performed in an anechoic chamber, where a person walked around the recording subject while rubbing two pieces of paper (see Methods for a detailed description). The recording was divided into 18 windows, each corresponding to a 20 degree part of a full circle. The number of windows was selected to match experimental parameters in [8, 10]. From each window 3000 epochs were drawn and each of them was encoded using the model. Computing histograms of coefficients *s* at each angular position *θ*, provided an estimate of conditional distributions *p*(*s*_*i*_∣*θ*). Panel A in Fig 7 displays a conditional histogram of coefficient *s* corresponding to the basis function pair depicted in Fig 4A.

Distributions of sparse coefficients revealed a strong dependence in the position of the sound source. As visible in the figure, the conditional mean of the distribution *p*(*s*_*i*_∣*θ*) traced by the red line varied in a pronounced way across all positions. By analogy to averaged firing rates of neurons, average unit responses at each position were further studied to understand the spatial sensitivity of basis functions. Mean vectors *μ*_*i*, *θ*_ were constructed for each second-layer unit by taking its average response at the sound source position *θ*. Each mean vector was shifted and scaled such that its minimum value was equal to 0 and the maximum to 1. Such transformation was analogical to physiological studies [8] and allowed for comparison with experimetally measured spatial tuning curves of auditory neurons, and for this reason scaled vectors *μ*_*i*_ will be referred to as model tuning curves in the remainder of the paper. In order to identify spatial tuning preferences, the population of model tuning curves was grouped into two clusters using the k-means algorithm. Obtained clusters consisted of 118 and 138 similar vectors. Tuning curves belonging to both clusters and revealing a strong correlation (∣*ρ*∣ > 0.75) with sound position are plotted in Fig 7C as gray lines. Cluster centroids (averages of all tuning curves belonging to a cluster) are plotted in black. Second layer units were tuned broadly—most of them were modulated by sound located at all positions surrounding the subject’s head. A clear spatial preference is visible—members of cluster 1 were most highly activated (on average) by sounds localized close to the left ear (*θ* ≈ −90°), while cluster 2 consisted of units tuned to the right ear (*θ* ≈ 90°). Very similar tuning properties of auditory neurons were identified in the cat’s auditory cortex [8]. Data from this study is plotted for comparison in the subfigure B of Fig 7. Neuronal recordings were performed in the right hemisphere and two panels depict two subpopulations of neurons. The larger contra- and the smaller ipsi-lateral one. It is important to note, that the notion of ipsi, and contra laterality is not meaningful in the proposed model, therefore one should compare shapes of model and experimental tuning curves, not the numerosity of units in each population or cluster.

**Fig 8:**
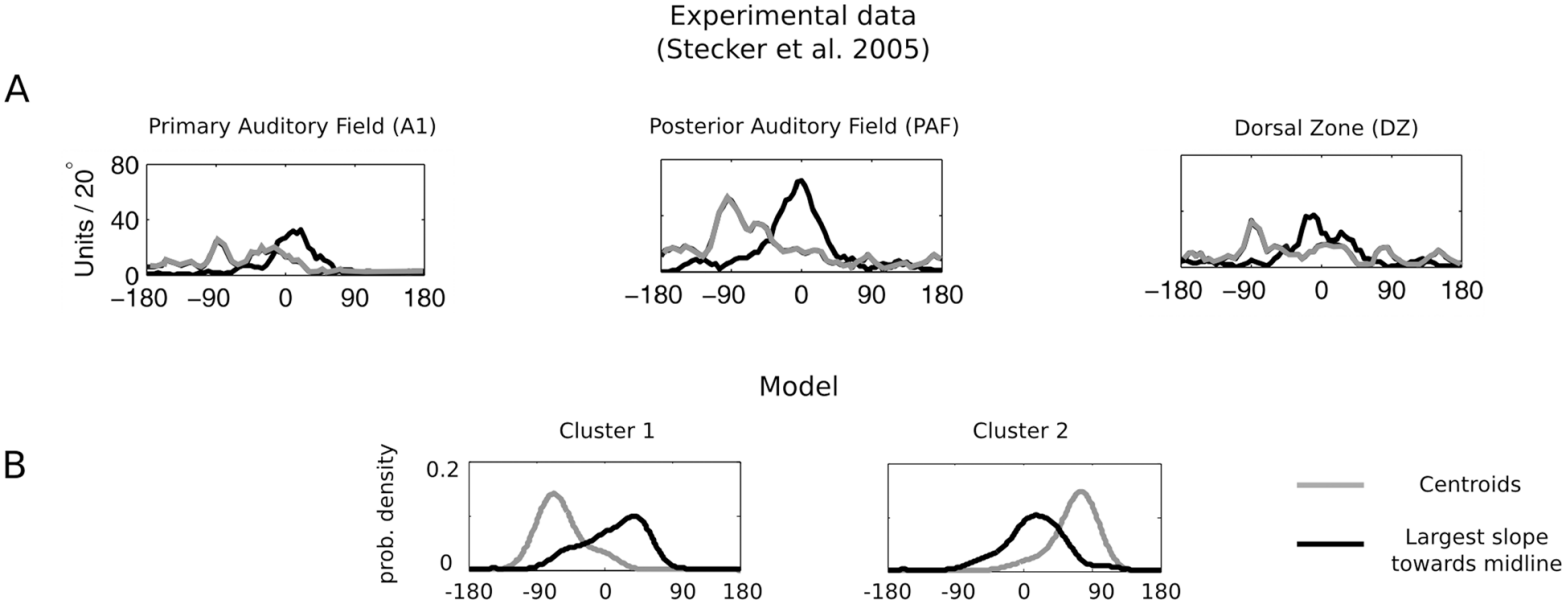
Distribution of tuning curve centroids and maximal slope positions in the model and experimental data. A) Histograms of positions of tuning curve centroids (gray) and maximal slopes towards the midline (black) measured experimentally in the auditory cortical areas from the cat. Figure modified from [8]. B) Distribution of the same features computed for model tuning curves belonging to each cluster.

Two major features of cortical auditory neurons responsive to sound position were observed experimentally: (i) tuning curve peaks were localized mostly at extremely lateral positions (opposite to each ear) and (ii) slopes of tuning curves were steepest close to the auditory midline. Both properties are visible in model tuning curves in Fig 7. However, in order to perform a more direct comparison between the model and experimental data, analysis analogous to the one described in [8] was performed. First, tuning curve centroids were computed. A centroid was defined as an average position, where the unit activation was equal to 0.75 or larger (see Methods). In the following step, the position of maximal slope towards midline was identified for each unit. This meant that for units tuned to the left hemifield (cluster 1) the position of the minimal slope value was taken, while the position of the maximal one was taken for units tuned to the right hemifield (cluster 2). In this way, the position of maximal sensitivity to changes in sound location was identified. Distributions of model centroids and maximal slope positions are depicted in Fig 8B. Centroids were distributed close to lateral positions, opposite in each cluster (−90° cluster 1, +90° cluster 2). Distribution peaks were located at positions close to each ear. No uniform tiling of the space by centroid values was present. At the same time, maximal slope values were tightly packed around the midline—peaks of their distributions were located precisely at, or very close to 0 degrees. This means that while the maximal response was on average triggered by lateral stimuli, the largest changes were triggered by sounds located close to the midline. Both properties were in good agreement with the experimental data reported in [8]. Fig 8A depicts in three panels centroid and slopes distributions measured in three different regions of cat’s auditory cortex—Primary Auditory Field (A1), Posterior Auditory Field (PAF) and Dorsal Zone (DZ). A close resemblance between the model and physiological data was visible.

It has been argued that while single neurons in the auditory cortex provide coarse spatial information, their populations form a distributed code for sound localization [8, 9, 9, 10]. Here, a decoding analysis was performed to verify whether similar statement can be made about the proposed model.

**Fig 9:**
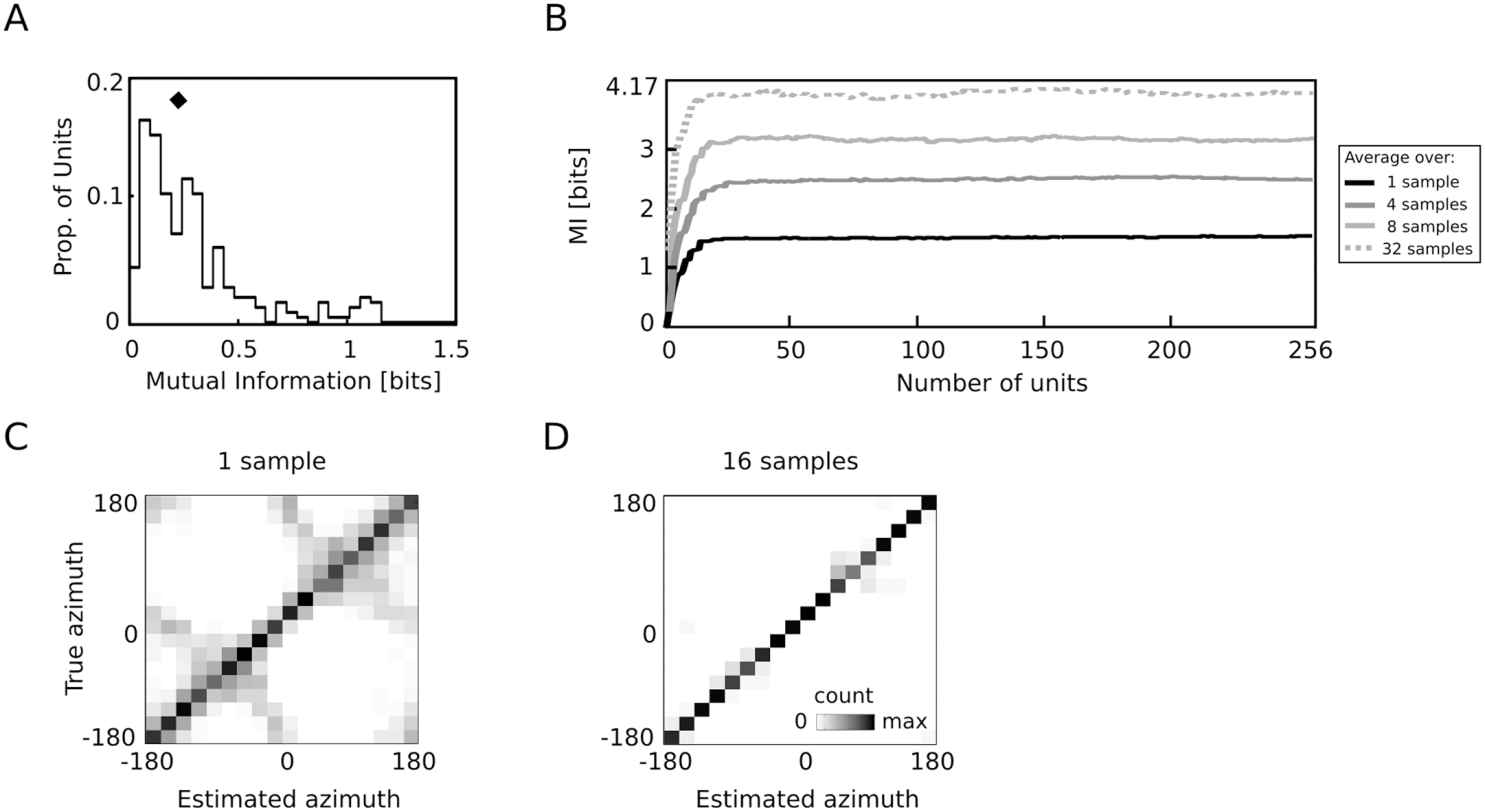
Population decoding analysis. A) Histogram of position-specific information carried by second layer sparse coefficients *s*. The diamond symbol marks the distribution median. B) Mutual information plotted as a function of the number of units used to decode the position. Colors of lines correspond to data averaged over different number of samples. The scale ends at 4.17 bits, which is the amount of information required to perform errorless decoding (log_2_(18) = 4.17) C) Confusion matrix for decoding of population responses from a single sample, and D) averaged across 16 samples.

A gaussian mixture model (GMM) was utilized as a decoder. The GMM modelled the marginal distribution of sparse coefficients as a linear combination of 18 gaussian components, each corresponding to a particular position of a sound source (i.e. the *θ* value). In the first part of the decoding analysis, single coefficients were used to identify the sound position. The GMM was fitted using the training dataset consisting of coefficient values *s*_*i*_ and associated position labels *θ*. In the testing stage, position estimates $\hat{\theta }$ were estimated (decoded) using unlabeled coefficients from the test dataset (see Methods section for a detailed description of the decoding procedure). For each of the coefficients, a confusion matrix was computed. A confusion matrix is a two-dimensional histogram of *θ* and $\hat{\theta }$ and can be understood as an estimate of the joint probability distribution of these two variables. Using a confusion matrix, an estimate of mutual information (i.e., the number of bits shared between the position estimate $\hat{\theta }$ and its actual value *θ*) was obtained. Fig 9A depicts histograms of information carried by each coefficient *s*_*i*_ about the sound source position, estimated as described above. A general observation is that single coefficients carried very little information about the sound location. The histogram peaks at a value close to 0.1 bits. Only few units coded approximately 1 bit of positional information. Even 1 bit, however, suffices merely to identify a hemifield, not to mention the precise sound position. As can be predicted from the broad shapes of the tuning curves, single second-layer units carried little spatial information. A similar result was obtained for neurons in different areas of the cats auditory cortex [12]. The amount of information about the sound position encoded by spike count of neurons in A1 and PAF regions has a distribution closely similar to that of model units (compare with the left panel of figure 11 in [46]). Spike count (which essentially corresponds to a firing rate) is a feature of a neuronal response most directly corresponding to coefficients *s* in the model described here. The median of mutual information estimated from model coefficients (marked by a diamond symbol in panel A) aligns well with the same quantity estimated from neuronal data, and is close to 0.2 bits [46]. Overall, physiological measurements and the behavior of the model were highly similar.

While single neurons did not carry much spatial information, the joint population activity was sufficient to decode the sound position [8–10, 46]. Therefore in the second step of the decoding analysis, multiple coefficients *s* were used to train and test the GMM decoder. Results of the population decoding are plotted in Fig 9B. The decoder was trained with a progressively larger number of second-layer units (from 1 to 256) and the mutual information was estimated from obtained confusion matrices. Each line in the plot depicts the number of bits as a function of the number of units used to perform decoding. Line colors correspond to the number of samples over which the average activity was computed. Broadly speaking, larger populations of second-layer units allowed for a more precise position decoding. As in the case of single units, averages over larger amounts of samples were also more informative—population activity averaged over 32 samples saturated amount of bits required to perform errorless decoding (4.17). Two confusion matrices obtained from raw population activity and an average over 16 samples are displayed in subfigures Fig 9C and 9D. In the former case, the decoder was mostly misclassifying sound positions within each hemifield. Averaging over 16 sound samples yielded an almost diagonal (errorless) confusion matrix. The decoding analysis allowed us to draw the conclusion that while single units carried very little spatial information, their population encoded source location accurately, consistent with experimental data.

**Fig 10:**
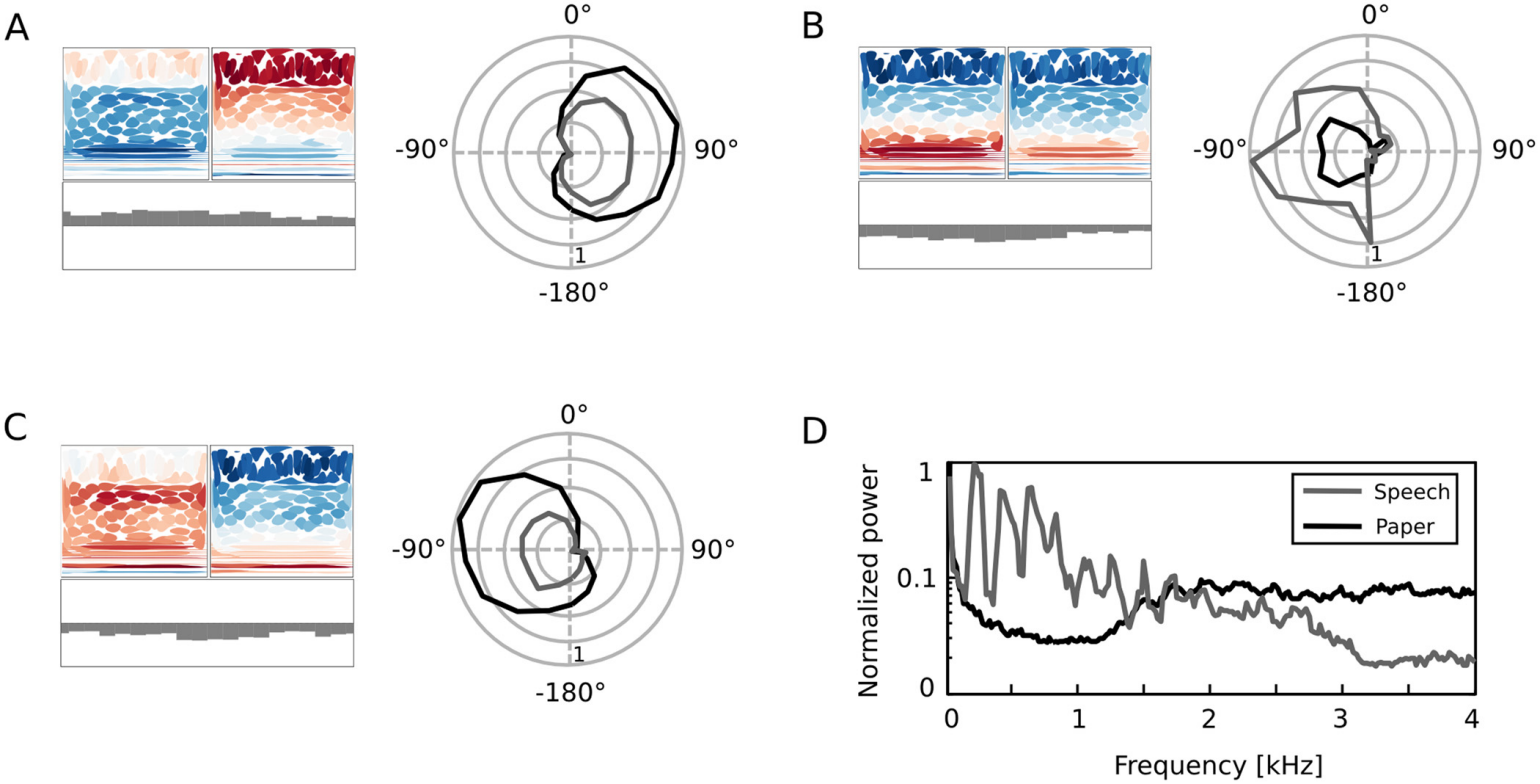
Comodulation of unit responses by sound position and identity. A)-C) Three representative second layer basis functions plotted with spatial tuning curves obtained using two different sounds—female speech (gray) and paper noise (black). D) Frequency spectra of both test sounds.

Second layer units achieved spatial tuning by assigning different weights to amplitudes in each ear, and to IPD values in different frequency channels. At the same time they encoded spectrotemporal features of sound, as depicted in Fig 4. Their activity should therefore be modulated by both sound position as well as its quality. Such comodulation is a prominent feature of the majority of cortical auditory neurons [1, 7]. In order to verify this, model spatial tuning curves were estimated with a second sound source, very different from a hiss created by rubbing paper—human speech (see Materials and Methods for details). Frequency spectra of both test stimuli are depicted in Fig 10D. Test sounds distributed their energy over non-overlapping parts of the frequency spectrum. While speech consisted mostly of harmonic peaks below 1.5 kHz, the paper sound was much more broadband and its energy was uniformely distributed between 1.5 and 4 kHz.

Panels A-C of Fig 10 depict three amplitude/IPD basis function pairs together with their spatial tuning curves estimated using different sounds. The spatial preference of depicted units (left or right hemifield) was predictable from their binaural composition. Each of them, however, was activated stronger by a stimulus, which spectrum matched better amplitude basis functions. Basis functions visible in panels A and C had a lot of energy accumulated in higher frequencies, therefore the paper sound activated them stronger (on average). Basis function B) was spectrally better corresponding to speech sounds, therefore speech was a preferred class of stimuli. This observation suggests that tuning curves i.e. position-conditional means *μ*_*i*, *θ*_ should be understood not as averages of coefficient ensembles conditioned only on the sound position *θ* but also on spectral properties of sound. When interpreting coefficients *s* as neuronal activity this means that spatial tuning curves would alter their shapes when the neuron is tested with two different sound sources. Taken together, one can state that the second-layer representation encoded position and identity of the stimulus in an interdependent fashion.

## Discussion

Previously proposed statistical models of natural acoustic stimuli focused predominantly on monaural sounds [22–25, 30, 38]. Studies modelling binaural stimuli were constrained to a limited representation—either IPDs [45] or spectrograms [31]. In contrast, the assumption behind the present work was that spatial sensitivity of cortical neurons is formed by fusing different cues. Therefore, in order to understand the role played by the auditory cortex in spatial hearing, the entire natural input processed by the auditory system was analyzed.

To this end, a novel probabilistic model of natural stereo sounds has been proposed. The model is based on principles of sparse, efficient coding—its task was to learn progressively less redundant representations of natural signal. It consisted of two hidden layers, each of them could be interpreted as an analogy to different stages of sound processing in the nervous system. The purpose of the first layer was to form a sparse, non-redundant representation of natural sound in each ear. By analogy to the cochlea, the encoding was supposed to extract and separate temporal information (i.e. phase) from the amplitude of the signal. In order to do so, a dictionary of complex-valued basis functions was adapted to short sound epochs. On top of the first model layer, which encoded sound in each ear independently, the second layer was trained. Its goal was to encode jointly amplitude and phase—two kinds of information crucial for sound localization, which may be fused together in higher stages of the auditory system. The higher-order representation captured spectrotemporal composition of the signal, by learning amplitude patterns of the first layer output as well as interaural disparities present in form of interaural phase and amplitude differences. It is important to stress that the model was fully unsupervised—at no point information about positions of sounds sources or the spatial configuration of the environment was accessible. Yet, when tested with a set of spatial sounds, activity of second layer units revealed strong dependence on sound position. Tuning curves describing relation between the sound position and model activity were in good correspondence with experimentally measured spatial tuning properties of cortical auditory neurons.

### A sparse representation of natural binaural sounds forms a panoramic population code for sound location

In mammals, the location of a sound is encoded by two populations of broadly tuned, spatially non-specific units [32]. This finding challenges initial expectations of finding a “labelled-line code” (i.e. a topographic map of neurons narrowly tuned to small areas of space). The “spatiotopic map” was expected by analogy to the tonotopic structure of the cortex, as well as the high localisation accuracy of humans and animals. Instead, it has been found that auditory cortical neurons within each hemisphere are predominantly tuned to far, contralateral positions. Peaks of observed tuning curves did not tile the auditory space uniformly, rather they were clustered around the two lateral positions. A prominent observed feature of cortical representation of sound location were slopes of the tuning curves. Regardless of the position of the tuning curve peak, slopes were steepest close to the interaural midline—the area where behavioral localisation acuity is highest [32]. From described observations, two prominent conclusions were drawn. Firstly, that the slope of tuning curves, not the distribution of their peaks determines spatial acuity [8, 32, 47, 48]. Secondly that sound position is encoded by distributed patterns of population activity, not single neurons [8–10]. It has been argued that these properties are a manifestation of a coding mechanism which evolved to specifically meet the demand of binaural hearing tasks [8, 32]. Here it is shown that crucial properties of cortical spatial tuning emerge in an unsupervised learning model, which learns a sparse representation of natural binaural sounds. The objective of the model was to code the stimulus efficiently (i.e. with a minimal redundancy within limits of applied transformations), while minimizing unit activity. Properties of the learned representation are therefore a reflection of stimulus statistics, not of any task-specific coding strategy (required for instance to localize sounds with the highest accuracy at the midline).

The position of the sound-generating object is a latent variable for the auditory system. It means that its value is not explicitly present in the raw stimulus—it has to be estimated. This estimation, (or inference) is a non-trivial task in the real acoustic environment, where sounds reaching ear membranes are a reflection of intricate auditory scenes. Sensory neurons perform transformations of those sound waveforms to reconstruct the spatial configuration of the scene. Therefore, in an attempt to understand cortical representation of space, it may be helpful to think what is the statistical structure of the naturally encountered binaural stimulus that the auditory system operates on. Sounds reaching the ear contain information about their generating sources, the spatial configuration of the scene, position and motion of the organism and the geometry of its head and outer ears.

Results obtained here suggest that the shapes of the model spatial tuning curves reflect regularities imposed on the sensory data by the filtering properties of the head. At lateral positions (directly next to the left or the right ear) there is no acoustic attenuation by the skull, hence sounds are loudest and least delayed. This in turn, elicits the strongest response in units preferring that side. When the sound is at a contralateral position, response is much weaker, due to the maximal head attenuation and largest delay. The curve connecting those two extrema is steepest in the transition area—at the midline. Since the auditory environment was uniformly sampled at both sides of the head, model units were clustered into two roughly equal subpopulations, basing on the shapes of their tuning curves. Clusters were symmetric with respect to each other—one tuned to to the left and the other to the right hemifield. This groupping is reminiscent of the “opponent-channel” representation of the auditory space, which has been postulated before [8, 32]. Present results provide a theoretical interpretation of this tuning pattern. They suggest that neuronal population which forms a sparse, efficient representation of natural stimuli would reveal two broadly tunned channels, when probed with sounds located at different positions.

It has been shown previously that IPD coding strategies in different species can be predicted from statistics of binaural sound [45]. Harper and McAlpine demonstrated that if the goal of the nervous system is to represent IPD values with the maximal possible accuracy (quantified by Fisher information) two populations of neurons tuned to opposite locations constitute an optimal representation of low-frequency IPDs. Their approach differs significantly from the one presented here. On the most abstract level, the authors of [45] assume that the purpose of IPD sensitive neurons is to maximize Fisher information, while here mutual information is the quantity implicitly maximized by the representation (although interesting relationships exist between those two measures [49]). Secondly, Harper and McAlpine limit their analysis to IPD statistics only—here entire binaural waveforms are modelled. Finally the current study does not assume any parameteric shape of tuning curves, nor make any other assumptions about physiology as is the case in [45]. The similarity of model responses and neuronal activity emerges from data statistics.

### Interdependent coding of spatial information and other features of the sound

There is an ongoing debate about the presence (or lack of thereof) of two-separate “what” and “where” streams in the auditory cortex [5]. The streams would separate spatial information from other sound features which determine its identity. An important prediction formed by this dual-stream hypothesis is that there should exist neurons selective to sound position and invariant to other aspects in the auditory cortex. While some evidence has been found supporting this notion [3, 4] it seems that at least in vast parts of the auditory cortex neural activity can be modulated by multiple features of sound such as pitch, timbre and location [1]. Neurons are sensitive to sound position (i.e. changing position affects their firing patterns), but not selective nor invariant to it. The majority of studies analyzing spatial sensitivity in the auditory cortex use a single class of sound and the source position is the only varying parameter. Therefore, despite initial efforts, the influence jointly exerted by sound quality and position on neuronal activity is not yet well understood.

The statistical model proposed here suggests that no dissociation of spatial and non-spatial information is necessary to either reconstruct the sound source or identify its position. The learned second-layer representation carries both kinds of information—about the sound quality (contained in the spectrotemporal structure of basis functions) and about spatial aspects (contained in the binaural amplitude weighting and IPD vectors). The learned code forms a “what is where” representation of the stimulus (i.e., those two aspects are represented interdependently). A manifestation of this fact is visible in different scaling of spatial tuning curves, when probed with two different sound sources. Such comodulation of neuronal activity by sound position and quality has been observed experimentally [1], which may suggest that recorded neurons form a sparse, efficient representation of binaural sound. An advantage of an interdependent “what is where” representation is the absence of the “feature binding problem”, which has to be solved if spatial information is processed independently. After separating the location of a source from its identity they would have to be fused at processing stages beyond the auditory cortex. A code similar to the one described here does not create such a problem. This idea goes in hand with results of a recent perceptual study [50]. Parise et al. demonstrated that the perception of sound source elevation is strongly influenced by its frequency. Furthermore they show that this relationship can be explained by adaptation to the joint distribution of natural sounds’ positions and spectra. This implies that the quality of the sound source as well as its spatial position are mutually dependent, and as such should be represented jointly, if the goal of the nervous system is to increase coding efficiency.

### Limitations and possible extensions

The model proposed in this work is a statistical one—it constitutes an attempt to describe functional, not anatomical modules of the auditory system. Rather than explicitly modelling stages of the auditory pathway, its goal is to approximate the distribution of natural binaural sounds. The behaviour of units in the highest layer reveals a strong resemblance to cortical auditory neurons in an abstract, information processing domain. In the mammalian auditory system the sound is processed in at least five anatomical structures before it reaches the cortex [51]. It is therefore almost certain that the stimulus is subjected to many more complex transformations than the ones proposed here. On the other hand, the fact that similiarities between cortical and model responses emerge despite this lack of detail, imply that the model may be capturing some aspects of information processing, as it happens in the real auditory system.

The relationship between abstract computational principles such as sparse coding and neurophysiology is an area of ongoing research [52–54]. An interesting extension of the present work would attempt to increase the level of biological detail, and see whether this allows formation of more refined experimental predictions. This could be done by implementing sparse coding computations using spiking neuron models, as it has been done in studies of the visual system (e.g. [52, 54]). The match between the model and biology could be also improved by including phenomena specific to the auditory system, such as the phase locking in the auditory nerve.

This study focuses predominantly on explaining the broad spatial tuning of cortical auditory neurons estimated by the analysis of firing rates. With progressively larger amounts of biological detail added to the model, one could attempt to explain other aspects of spatial information encoding. For instance, the notion of spike timing does not exist in the approach proposed here, while temporal spike patterns of cortical neurons seem to carry relevant spatial information [9, 10, 46]. Moreover, as mentioned in the results section, the concept of contra- and ipsilaterality is spurious for high-layer model units since they are not associated with any anatomical locus (left or right hemisphere). Overrepresentation of the contralateral ear is an interesting feature of panoramic population codes [8], which is also not addressed by the present work. Further exploration of the relationship between specific biological observations and spatial information processing constitutes a possible goal for future research.

It is highly likely that the main result of this study (i.e., spatial tuning properties of the binaural sound representation) could be reproduced by replacing the first layer with a different sort of spectrotemporal signal representation. It would not necessarily have to be the sparse, efficient encoding of sound epochs. A spectrogram could be a candidate signal, although it has been demonstrated that a sparse code of relatively long binaural spectrogram chunks generates features of very different spatial tuning [31]. In this work, for the sake of theoretical consistency, both layers were learned using the same principles and statistical assumptions—sparse factorial coding.

The data used for comparisons originated from studies of cat auditory cortex ([8, 46]). Since statistics of the binaural signal are affected by the geometry of ears and the head of the organisms, one could argue that model trained on binaural recordings performed by a human should not be compared with cat physiology. As long as detailed features of neuronal tuning to a sound position may vary across those species, tuning patterns highly similar to those of the cat have been observed in the auditory cortex of primates [55, 56]. Overall, the cortical representation of sound position seems to be highly similar across mammals [32].

Finally, in the current study a binaural recording of only a single auditory scene was used to train the model. Even though the recording included many types of sound—ambient environmental noises, transient cracks and clicks and harmonic structures such as the human speech, it did not include many other possible sources (for instance animal vocalizations). The recording included also only a narrow range of other parameters which characterize natural auditory scenes, such as reverberation. Analysis of longer recordings performed in different environmental settings may generate more diverse results and additional insights. One should note however, that certain properties of the learned representation (such as the tradeoff in the spectrotemporal modulation) seem to be a general proprerty of natural sounds as such and remain invariant to a specific dataset [25, 40]. Basing on this observation one may expect that units revealing similar spatial tuning can be learned from recordings of numerous, diverse sets of natural sounds.

### Conclusion

Taken together, this paper proposes a candidate theoretical mechanism explaining how neurons in the auditory cortex represent spatial information. This model allows us to speculate they do not have to implement any task-dependent strategy. Instead, their behavior can be explained by sparse coding—a statistical model which has succesfully predicted properties of multiple other sensory systems [18, 21]. Taking a broad perspective, (as suggested by Barlow in his later work [57, 58]) this means that redundancy reduction by sparse coding can be used by the brain to identify sensory data patterns allowing sucesful interaction with the environment.

## Methods

### Ethics statement

Sound recordings received approval of the Ethics Council of the Max-Planck Society. Human participants provided a written consent to participate in recordings.

### Binaural recordings

Sounds used to train and test the model were recorded using Soundman OKM-II binaural microphones placed in the ear channels of a human subject, whose head circumference was 60 cm. While recording training sounds, the subject walked freely in a wooded area accompanied by another person who spoke rarely. In this way, collected data included transient and ambient environmental sounds as well as harmonic speech. The binaural composition of sound was affected by spatial configuration of the environment and motion patterns of the recording subject. The recording used to train the model was 60 seconds long in total. Binaural recordings are availible in the supplementary material of [59].

Test recordings used to map the spatial tuning of second-layer units was performed in an anechoic chamber at the Department of Biology, University of Leipzig. The same recording subject was seated in the middle of the chamber. A female speaker walked at a constant pace following a circular path surrounding the recording subject. While walking she counted out loud. This was repeated four times. The second test recording was performed in a similar fashion, however instead of speaking the walking person rubbed two pieces of cardboard against each other, generating a broadband sound. To estimate the conditional distribution of sparse coefficients given the position and identity of the sound, test recordings were divided into 18 intervals, each corresponding to the same position on a circle.

All recordings were registered in an uncompressed wave format at 44100 Hz sampling rate. Prior to training the model, sounds were downsampled to 8000 Hz. Test recordings are availible in the supplementary material (S1, S2, S3, S4, S5, S6, S7, S8 Files).

### Learning and inference

The goal of the learning procedure was to estimate first- (*A*), and second- layer basis functions (*B*, *ξ*). This was done using a two-step approach. Firstly maximum a posteriori (MAP) estimates of model coefficients (*z* in the first layer, *s* and *w* in the second) were inferred via gradient descent [18, 33]. Secondly, a gradient update on basis functions was perormed using current coefficient estimates. Those two steps were consecutively iterated until the model converged.

A dictionary of complex-basis functions in the first layer was created by first, training a standard sparse code of sound epochs *x* ∈ ℝ^*T*^:

$$\begin{array}{c} x_{t}=\sum_{i=1}^{T}c_{i}\Theta_{i,t}+\eta \end{array}$$ (18)

The negative log-posterior of this model was:

$$\begin{array}{c} E_{s}\left(x,c,\Theta \right)\propto \frac{1}{\sigma^{2}}\sum_{t=1}^{T}{\left(x_{t}-{\hat{x}}_{t}^{s}\right)}^{2}+\lambda \sum_{i=1}^{T}S\left(c_{i}\right) \end{array}$$ (19)

where ${\hat{x}}_{t}^{s}=\sum_{i=1}^{T}c_{i}\Theta_{i,t}$ is the reconstruction of the data vector. Corresponding gradients over linear coefficients *c* and basis functions Θ were given by:

$$\begin{array}{c} \frac{\partial }{\partial c_{i}}E_{s}\propto -\frac{2}{\sigma^{2}}\sum_{j=1}^{T}\Theta_{j,t}\left(x_{t}-{\hat{x}}_{t}^{s}\right)+2\lambda \frac{c_{i}}{\log(1+c_{i}^{2})} \end{array}$$ (20)

$$\begin{array}{c} \frac{\partial }{\partial \Theta_{i,t}}E_{s}\propto -\frac{2}{\sigma^{2}}\sum_{t=1}^{T}c_{i}\left(x_{t}-{\hat{x}}_{t}^{s}\right) \end{array}$$ (21)

Learned basis functions Θ_*i*_ were used as real vectors $A_{i}^{ℜ}$ and extended with their Hilbert transforms. Such complex basis function dictionary was used to encode monaural sound epochs. Gradients of Eq 5 over phase *ϕ*_*i*_ and amplitudes *a*_*i*_ of complex coefficients *z*_*i*_ were equal to:

$$\begin{array}{c} \frac{\partial }{\partial a_{i}}E_{1}\propto -\frac{2}{\sigma^{2}}\sum_{t=1}^{T}(\cos\phi_{i}A_{i,t}^{ℜ}+\sin\phi_{i}A_{i,t}^{ℑ})\left(x_{t}-{\hat{x}}_{t}\right)+2\lambda \frac{a_{i}}{\log(1+a_{i}^{2})} \end{array}$$ (22)

$$\begin{array}{c} \frac{\partial }{\partial \phi_{i}}E_{1}\propto -\frac{2}{\sigma^{2}}\sum_{t=1}^{T}a_{i}\left(A_{i,t}^{ℑ}\cos\phi_{i}A_{i,t}^{ℑ}-A_{i,t}^{ℜ}\sin\phi_{i}A_{i,t}^{ℜ}\right)\left(x_{t}-{\hat{x}}_{t}\right) \end{array}$$ (23)

The second layer of the model was trained after the first layer converged, and cofficient values *z* were inferred for all training data samples. The higher order encoding formed by coefficients *s* as well as the scaling factor *w* was inferred via gradient descent on function *E*_2_ (Eq 13):

$$\begin{array}{c} \frac{\partial }{\partial s_{i}}E_{2}\propto -\frac{2}{\sigma_{2}^{2}}\sum_{n=1}^{2\times T}B_{i,n}\left(a_{n}-{\hat{a}}_{n}\right)+\kappa \left|w\right|\sum_{m=1}^{P}\sin\left(\Delta \phi_{m}-{\hat{\Delta \phi }}_{m}\right)\xi_{i,m}+2\lambda_{2}\frac{s_{i}}{\log(1+s_{i}^{2})} \end{array}$$ (24)

$$\begin{array}{c} \frac{\partial }{\partial w_{i}}E_{2}\propto \kappa \frac{w}{{\left|w\right|}^{2}}\sum_{m=1}^{P}{\hat{\Delta \phi }}_{m}\sin\left(\Delta \phi_{m}-{\hat{\Delta \phi }}_{m}\right)+\lambda_{w}[{\left(\frac{1}{\alpha }\right)}^{\beta }\beta w{\left|w\right|}^{\beta -2}] \end{array}$$ (25)

The gradients steered sparse coefficients *s* to explain amplitude and phase vectors *a* and Δ*ϕ* while preserving maximal sparsity. Simultaneously the multiplicative factor *w* was adjusted to appropriately scale the estimated vector $\hat{\Delta \phi }$.

Finally, learning rules for second-layer dictionaries were given by:

$$\begin{array}{c} \frac{\partial }{\partial B_{i,k}}E_{2}\propto -\frac{2}{\sigma_{2}^{2}}s_{i}\left(a_{k}-{\hat{a}}_{k}\right) \end{array}$$ (26)

$$\begin{array}{c} \frac{\partial }{\partial \xi_{i,k}}E_{2}\propto s_{i}\kappa \left|w\right|\sin\left(\Delta \phi_{k}-{\hat{\Delta \phi }}_{k}\right) \end{array}$$ (27)

Altogether 75000 epochs of binaural sound were used to train the model. Each of them was *T* = 128 samples long, which corresponded to 16 ms. Both layers were trained separately. Before training the first layer, Principal Component Analyis was perfomed and 18 out of 128 principal components were rejected, which corresponded to low pass filtering the data. Left and right ear sound epochs were shuffled together to create a 150000 sample training dataset for the first layer. The first layer sparsity coefficient *λ* was set to 0.2. Noise variance *σ* ^2^ was equal to 2. The sparse coding algorithm converged after 200000 iterations.

A complex-valued dictionary was created by extending the real valued one with Hilbert-transformed basis functions. Amplitude and phase vectors *a* and *ϕ* were inferred for each sample using 20 gradient steps. Amplitude vectors were concatenated and transformed with a logarithmic function, and IPD vectors Δ*ϕ* were computed by substracting left ear phase vectors *ϕ*_*L*_ from right ear ones *ϕ*_*R*_. The second layer was trained by performing 250000 gradient updates on basis functions *B* and *ξ*. The amplitude sparsity coefficient *λ*_2_ was set to 1. The *λ*_*w*_ parameter was set to 0.01 and the noise variance $\sigma_{2}^{2}$ as well as the von Mises concentration parameter *κ* were set to 2.

Numerical values of the prior-controlling parameters *λ*, *λ*_2_, *λ*_*w*_ as well as noise parameters *σ*, *σ*_2_, *κ* were set empirically in this study. By running simulations with multiple parameter settings it has been found that due to the presence of a strong environmental noise in the training recording, noise variances *σ*, *σ*_2_ and the von Mises concentration parameter *κ* should be relatively large in order to achieve convergence. Sparsity of the high layer representation was set to be larger than that of the first layer in order to mimic the biological intuition that neural responses in the ascending auditory pathway become progressively less redundant and sparser [20, 60]. It has been found however, that the exact value of sparsity paramaters did not affect the spectrotemporal properties, nor the spatial tuning of the second layer units strongly. The *λ*_*w*_ parameter which controls the strength of the prior over the multiplicative factor *w* was set to be relatively small. Otherwise the *w* prior term in the Eq 16 became too strong and dominated learning, preventing the convergence. More principled and theoretically sound ways of parameter selection are possible. One could ask what are the natural noise levels and sparsity values of the training data by specifying them as hyperparameters of the model and learning the appropriate values. Also the number of basis functions at each level could be treated as a parameter and estimated from the data, not chosen ad-hoc. After extending the model in this way, the choice of the correct parameter setting could be performed by cross-validation or Bayesian model selection (as in [61]).

### Computation of modulation spectra of second-layer basis functions

Spectrograms of amplitude basis functions *B*_*i*_ were computed by combining spectrograms of real, first layer basis functions $A_{n}^{ℜ}$, linearly weighted by a corresponding weight exp(*B*_*i*, *n*_). First layer spectrograms were computed using *T* = 29 windows, each 16 samples (0.002 second) long, with a 12 sample overlap. Altogether, *F* = 128 logarithmically-spaced frequencies were sampled. A two-dimensional fourier transform of each spectrogram was computed using the matlab built-in function fft2. The amplitude spectrum of obtained transform is called the Modulation Transfer Function (MTF) of each second layer feature [40]. The center of mass i.e. the point $\left(C_{S,i}^{f},C_{S,i}^{t}\right)$ of each monaural part (*S* ∈ {*L*, *R*}) of basis functions *B*_*i*_ was computed in the following way:

$$\begin{array}{c} C_{S_{i}}^{t}=\sum_{t}t\sum_{f}MTF\left(B_{S,i}\right) \end{array}$$ (28)

$$\begin{array}{c} C_{S_{i}}^{f}=\sum_{f}f\sum_{t}MTF\left(B_{S,i}\right) \end{array}$$ (29)

where *t* and *f* are time and frequency respectively.

### Estimation of spatial tuning curves

To estimate conditional distribution of sparse coefficients given the position and identity of the sound, test recordings of a sound source (either speech, or rubbed paper) moving around the recording subject were used. Each source circled the recording person 4 times resulting in 4 recordings. Each of them was divided into 18 intervals. Intervals corresponding to the same area on the circle were joined together across all recordings. For each out of 18 sound positions 3000 random sound chunks were drawn and encoded by the model. Position-conditional ensembles were then used to compute conditional histograms. Conditional mean vectors *μ*_*i*, *θ*_ were computed by averaging all values of coefficient *s*_*i*_ at position *θ*. Mean vectors were mapped to a [0, 1] interval by adding the absolute value of a minimal entry and dividing it by the value of the maximum. For plotting purposes in Fig 10, endings of tuning curves were connected if values at −180° and 180° were not exactly equal.

### Decoding of stimulus position

The decoding analysis was performed using *K* second-layer sparse coefficients *s* averaged over *D* of samples. The response vectors *d* ∈ ℝ^*K*^ were therefore formed as:

$$\begin{array}{c} d=\frac{1}{D}\sum_{i=1}^{D}s_{\left\{1,\ldots ,K\right\}} \end{array}$$ (30)

Such averaging procedure can be interpreted as an analogy to computation of firing rates in real neurons.

The marginal distribution of response coefficients *d* over all 18 sound positions *θ* ∈ {−180°, −160°, …,160°,180°} was equal to:

$$\begin{array}{c} p\left(d\right)=\sum_{\theta }p\left(d|\theta \right)p\left(\theta \right) \end{array}$$ (31)

where each conditional *p*(*d*∣*θ*) was a *K*-dimensional Gaussian distribution with class specific mean vector *μ*_*θ*_ and covariance matrix *C*_*θ*_:

$$\begin{array}{c} p\left(d|\theta \right)=𝓝\left(\mu_{\theta },C_{\theta }\right) \end{array}$$ (32)

The prior over class labels *p*(*θ*) was uniformly distributed i.e. $p(\theta_{i})=\frac{1}{18}$ for each *i*.

The decoding procedure iterated over all class labels and returned the one, which maximized the likelihood of the observed data vector. Out of the entire dataset, 80% was used to train the model and remaining 20% to test and estimate the confusion matrix.

Confusion matrix *M* was a joint histogram of a decoded and true sound position $\hat{\theta }$ and *θ*. After normalization, it was an estimate of a joint probability mass function $p(\hat{\theta },\theta )$. Mutual information was estimated from each confusion matrix as:

$$\begin{array}{c} MI\left(\hat{\theta }\;\theta \right)=\sum_{\hat{\theta }}\sum_{\theta }p\left(\hat{\theta },\theta \right)\log_{2}(\frac{p(\hat{\theta },\theta )}{p\left(\hat{\theta }\right)p\left(\theta \right)}) \end{array}$$ (33)

## Supporting Information

S1 File Recording of a test sound source (paper hiss) moving around the recording subject. The recording was used to estimate spatial tuning curves of model units. The sound source circles the recording subject in a clockwise direction.
(WAV)

S2 File Recording of a test sound source (paper hiss) moving around the recording subject. The recording was used to estimate spatial tuning curves of model units. The sound source circles the recording subject in a clockwise direction.
(WAV)

S3 File Recording of a test sound source (paper hiss) moving around the recording subject. The recording was used to estimate spatial tuning curves of model units. The sound source circles the recording subject in a clockwise direction.
(WAV)

S4 File Recording of a test sound source (paper hiss) moving around the recording subject. The recording was used to estimate spatial tuning curves of model units. The sound source circles the recording subject in a clockwise direction.
(WAV)

S5 File Recording of a test sound source (female voice) moving around the recording subject. The recording was used to estimate spatial tuning curves of model units. The sound source circles the recording subject in a clockwise direction.
(WAV)

S6 File Recording of a test sound source (female voice) moving around the recording subject. The recording was used to estimate spatial tuning curves of model units. The sound source circles the recording subject in a clockwise direction.
(WAV)

S7 File Recording of a test sound source (female voice) moving around the recording subject. The recording was used to estimate spatial tuning curves of model units. The sound source circles the recording subject in a clockwise direction.
(WAV)

S8 File Recording of a test sound source (female voice) moving around the recording subject. The recording was used to estimate spatial tuning curves of model units. The sound source circles the recording subject in a clockwise direction.
(WAV)
