# Correction: The Opponent Channel Population Code of Sound Location Is an Efficient Representation of Natural Binaural Sounds

**DOI:** 10.1371/journal.pcbi.1004377

**Published:** 2015-08-19

**Authors:** The PLOS Computational Biology Staff

The corresponding author’s email address is incorrect. The correct address is: wiktor.mlynarski@gmail.com
